# Myelodysplastic neoplasms dissected into indolent, leukaemic and unfavourable subtypes by computational clustering of haematopoietic stem and progenitor cells

**DOI:** 10.1038/s41375-024-02203-z

**Published:** 2024-03-08

**Authors:** Margot F. van Spronsen, Sofie Van Gassen, Carolien Duetz, Theresia M. Westers, Yvan Saeys, Arjan A. van de Loosdrecht

**Affiliations:** 1 Department of Haematology, Amsterdam UMC, Vrije Universiteit Amsterdam, Cancer Centre Amsterdam, Amsterdam, Netherlands; 2 VIB Inflammation Research Centre, Ghent University, Ghent, Belgium; 3 Department of Applied Mathematics, Computer Science and Statistics, Ghent University, Ghent, Belgium

**Keywords:** Translational research, Myelodysplastic syndrome

## Abstract

Myelodysplastic neoplasms (MDS) encompass haematological malignancies, which are characterised by dysplasia, ineffective haematopoiesis and the risk of progression towards acute myeloid leukaemia (AML). Myelodysplastic neoplasms are notorious for their heterogeneity: clinical outcomes range from a near-normal life expectancy to leukaemic transformation or premature death due to cytopenia. The Molecular International Prognostic Scoring System made progress in the dissection of MDS by clinical outcomes. To contribute to the risk stratification of MDS by immunophenotypic profiles, this study performed computational clustering of flow cytometry data of CD34^+^ cells in 67 MDS, 67 AML patients and 49 controls. Our data revealed heterogeneity also within the MDS-derived CD34^+^ compartment. In MDS, maintenance of lymphoid progenitors and megakaryocytic-erythroid progenitors predicted favourable outcomes, whereas expansion of granulocyte-monocyte progenitors increased the risk of leukaemic transformation. The proliferation of haematopoietic stem cells and common myeloid progenitors with downregulated CD44 expression, suggestive of impaired haematopoietic differentiation, characterised a distinct MDS subtype with a poor overall survival. This exploratory study demonstrates the prognostic value of known and previously unexplored CD34^+^ populations and suggests the feasibility of dissecting MDS into a more indolent, a leukaemic and another unfavourable subtype.

## Introduction

Myelodysplastic neoplasms (MDS) are age-related haematopoietic disorders that are characterised by hypercellular, dysplastic bone marrow (BM) and peripheral blood (PB) cytopenia [1, 2]. As these features are not disease-specific, MDS comprise biological and clinical heterogeneity. Although MDS are thought to originate from the haematopoietic stem cell (HSC), abnormalities have been identified within the BM microenvironment and immune system [3–8]. Moreover, MDS patients’ outcomes range from a stable disease with a near-normal life expectancy and unrelated death (∼40% of patients) to the transformation towards acute myeloid leukaemia (AML, ∼25%) or BM failure with death due to complications of cytopenia (∼30%) [9]. As a result of heterogeneity, the diagnosis may be challenging due to overlapping entities while MDS patients’ clinical care relies on the accuracy of diagnostic and prognostic models [10–12]. These models tend to stratify MDS patients by estimating the overall and leukaemia-free survival based on the risk of all-cause mortality and a composite endpoint consisting of all-cause mortality and leukaemic progression [13, 14]. Although these endpoints may reflect dissimilar pathogenesis, no models have been developed that discriminate between unfavourable MDS due to AML or BM failure.

Recent insights into the genetic landscape of MDS resulted in the development of the Molecular International Prognostic Scoring System (IPSS-M), the World Health Organization (WHO) 2022 classification and the International Consensus Classification (ICC) [14–16]. Although genotype-phenotype correlations are currently the basis for MDS classifications, oncogenic abnormalities are not apparent in every MDS patient [17, 18]. Moreover, about 20% of the MDS patients with a very low-risk, low-risk or moderately low-risk following the IPSS-M may progress to AML within a few years [14]. Flow cytometric detection of leukaemia-associated immunophenotypes (LAIPs) of progenitor cells has complementary value to molecular profiling in AML [19]. Recently, we demonstrated that CD34^+^CD38^−^ stem cells expressing abnormal immunophenotypes similar to LAIPs, so-called immunophenotypic aberrant HSCs (IA-HSCs), predict leukaemic progression in MDS [20]. While manual gating strategies are still the standard, there is growing interest in the use of unsupervised analyses to generate an unbiased view of the haematopoietic system. This raises the question as to whether computational analysis of immunophenotypic data can identify haematopoietic populations that are beneficial in diagnosing and classifying MDS.

Here, we applied the unsupervised clustering algorithm FlowSOM to flow cytometry datasets containing BM specimens measured with the leukaemia-stem cell (LSC) tube [21, 22]. The LSC tube is an eight-colour assay designed at our laboratory for flow cytometric detection of LSCs in AML. We recently used this tube to detect IA-HSCs in MDS patients by manual gating [20]. In the current study, we used the same flow cytometry data for the computational analysis of haematopoietic stem and progenitor cells (HSPCs). We revealed that the heterogeneity of MDS extends to the immunophenotype of HSPCs and linked immunophenotypic signatures to MDS patients’ clinical characteristics and outcomes. In this way, we present an example of a dissection of MDS into a more indolent, a leukaemic and another unfavourable subtype.

## Methods

### Study cohort

The flow cytometry datasets were obtained from BM samples from cytopenic patients suspected of MDS and AML referred between 2014 and 2020 to our university hospital, Amsterdam UMC, location Vrije Universiteit Amsterdam. In total, 183 samples were selected, including MDS patients (*n* = 67), AML patients (*n* = 67), pathological controls (PCs, *n* = 39) and normal bone marrows from cardiothoracic surgery patients after written informed consent (NBMs, *n* = 10) (Fig. S1, Table S1). This work is an extension of a previous publication using the same flow cytometry data from MDS patients, PCs and NBMs [20]. This study was conducted following the Helsinki Declaration and approved by the Medical Ethics Committee of the Amsterdam UMC, location Vrije Universiteit Amsterdam (research ethics protocols: VUmc 2014-100, VUmc 2019-3448).

### Flow cytometry

Flow cytometry was performed using the LSC tube, designed to detect LSCs in AML (Table S2) [21]. This eight-colour assay combines CD7, CD11b, CD22, CD56, CD366 (TIM3) and CD371 (Clec12a) into the PE channel (further referred to as “Combi”) because of their absence on normal stem cells and includes CD45, CD34, CD38, CD33, CD44, CD45RA and CD123 in separate channels. Samples were not selected based on the immunophenotypic profile. Experimental procedures were carried out following standardised protocols to reduce technical noise (Supplementary Information (SI): Sample preparation, Flow cytometry).

### Computational analysis

Data were manually pre-gated to select mononuclear cells (MNCs) (Fig. S2) and subjected to pre-processing steps to reduce technical noise (SI: Data pre-processing). Pre-processed files were down-sampled to a maximum of 0.5 ∙ 10^6^ MNCs per file and aggregated into a dataset of 81 ∙ 10^6^ MNCs. To identify HSPCs, FlowSOM was applied to the MNC dataset using CD34, CD45 and scatter properties as input for cell clustering [22]. Two major CD34^+^ metaclusters were identified. All cells within the CD34^+^ metaclusters were selected, aggregated into a dataset of 16 ∙ 10^6^ CD34^+^ cells (Fig. S3) and subjected to FlowSOM again, discriminating distinct HSPC subsets based on all markers apart from the Combi channel. No clustering was observed on potential batch effects (Fig. S4). The FlowSOM metaclusters, hereafter termed populations, formed the basis for downstream analysis. Variance within population frequencies across samples was explored using principal component analyses.

### Statistical analysis

Chi-square and Mann-Whitney U- or Kruskal-Wallis tests were applied for testing categorical data in contingency tables and numerical data following a non-normal distribution, respectively. Univariate survival analysis was performed using the Kaplan-Meier method with the log-rank test for statistical comparison. The leukaemia free survival (LFS), event-free survival (EFS) and overall survival (OS) times were defined as the number of months from the date of BM sampling until the date of leukaemic transformation, disease progression defined as a blast increase of ≥ 5%, and all-cause mortality, respectively. Patients undergoing induction chemotherapy or stem cell transplantation were censored at the date of treatment start. Confidence intervals (CI) with 95% coverage were used and two-sided *P*-values ≤ 0.05 were considered statistically significant. Analyses were conducted with the statistical software R version 3.6 and the Statistical Package for the Social Sciences version 28.

## Results

### FlowSOM identifies distinct CD34^+^ subsets within MNCs from normal and neoplastic bone marrows

**Fig. 1:**
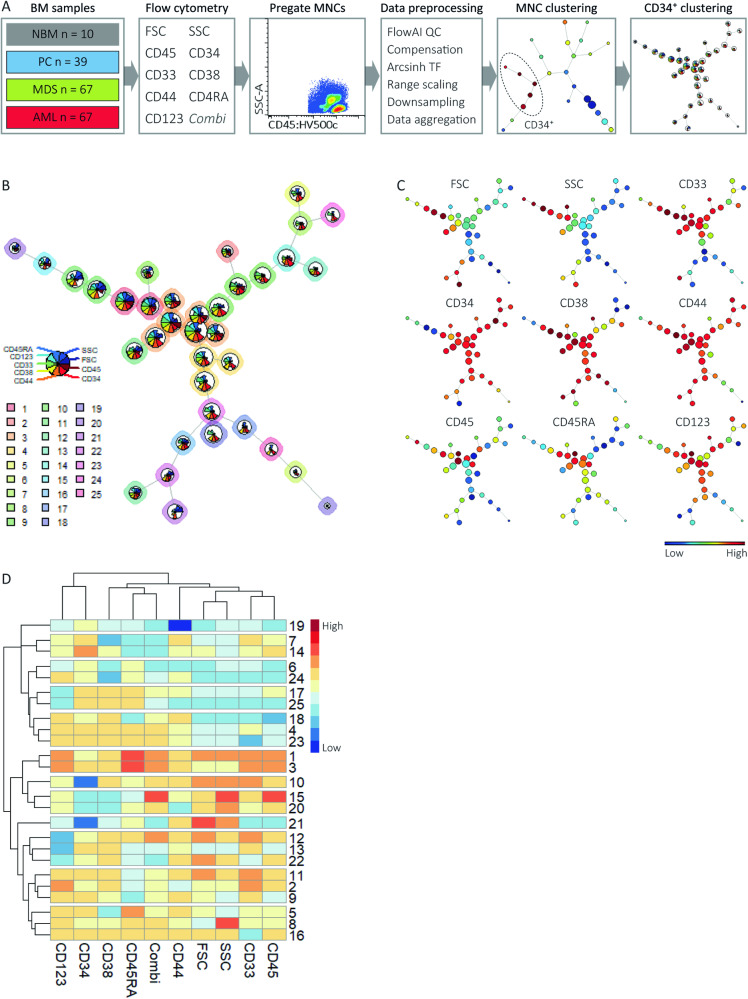
FlowSOM analysis of the CD34^+^ cell compartment from MDS patients and controls. **A** Experimental outline. BM samples from MDS patients (*n* = 67) and controls, including NBMs (*n* = 10), PCs (*n* = 39) and AML patients (*n* = 67), were selected. Flow cytometry was performed using the LSC tube. The MNC compartment was manually pre-gated and subjected to data pre-processing (SI: Data pre-processing). Pre-processed fcs files were aggregated and subjected to FlowSOM, using scatter properties, CD34 and CD45 as input for cell clustering. Two populations (divided over 5 clusters) with high CD34 expression were selected and subjected to FlowSOM again using scatter properties and all markers apart from those combined within the PE-channel to identify distinct HSPC subsets. **B** FlowSOM tree of CD34^+^ cells. The background colour of the nodes (*n* = 36) indicates their population (*n* = 25). The height of the plot pie visualises the expression of the surface markers and the scatter properties. The size of the nodes is proportional to the fraction of cells mapped to the node. **C** FlowSOM trees coloured by the median expression of indicated markers and scatter properties. The Combi channel was not used as input for cell clustering and is therefore not shown. Note that the CD34 and CD45 expressions are relative to the pre-gated CD45^dim^ CD34^+^ HSPC compartment. **D** Heatmap summary of scatter properties and marker expressions for each of the CD34^+^ populations. Each row represents one of the 25 populations. AML acute myeloid leukaemia, MDS myelodysplastic neoplasms, NBM normal bone marrow, PC pathological controls, QC quality control, TF transformation, MNC mononuclear cells, FSC forward scatter, SSC side scatter.

This study comprised 67 MDS patients covering distinct categories within the WHO and IPSS-R classification (Table 1). Sixty-seven AML patients, 39 PCs and 10 age-matched NBMs served as controls (Table S2). Application of FlowSOM on MNCs from all subjects using CD45, CD34 and scatter properties as input for clustering revealed distinct populations (Fig. 1A), further referred to by Roman numerals in square brackets [I-XII]. The 12 MNC populations included CD34^+^ progenitors [IV,VIII], CD34^-^ progenitors [II], SSC^low^ granulocytes [III], monocytes [V-VII], lymphocytes [I,IX] and erythroid cells [X] (Fig. S5). Application of FlowSOM on selected and aggregated 16 ∙ 10^6^ CD34^+^ progenitors [IV,VIII] using scatter properties and marker expressions apart from those combined within the PE-channel (Fig. 1B, C) resulted into the identification of 25 CD34^+^ populations, further referred to by numbers in square brackets [1–25] (Fig. 1D). For interpretation purposes, we labelled the CD34^+^ populations automatically and manually using Marker Enrichment Modelling and biaxial dotplots, respectively (Table 2). Seven populations contained CD38^-^ stem cells, including HSCs [7], CD45^low^ HSCs [24], CD44^dim/^^−^ HSCs [6,19,20], LSCs [5,15], and three populations contained CD38^dim^ HSCs [8,13,14]. Remaining populations included, amongst others, common lymphoid progenitors (CLPs) [17], common myeloid progenitors (CMPs) [2,9,11,18], megakaryocyte-erythroid progenitors (MEPs) [22] and granulocyte-monocyte progenitors (GMPs) [1,3,4]. In short, these data show that FlowSOM can distinguish well-known and more uncommon CD34^+^ subsets in normal and neoplastic BMs.

**Table 1:** MDS patient characteristics.

Characteristic	MDS (*n* = 67)	Characteristics	MDS (*n* = 67)
**Sex**, *n* (%)		**Age**	
Male	51 (76)	Median (range)	70 (30–89)
Female	16 (24)		
**Cell counts**, median (range)		**IPSS-R**, *n* (%)	
Haemoglobin (g/dL)	9.7 (6.0–15)	Very low	9 (13.4)
Platelets (·10^9^/L)	97 (10–754)	Low	29 (43.3)
Neutrophils (·10^9^/L)	2.4 (0–10)	Intermediate	11 (16.4)
White blood cells (·10^9^/L)	4.7 (0.5–35)	High	8 (11.9)
PB blasts (%)	0 (0–8)	Very high	8 (11.9)
BM blasts (%)	2 (0–17)	Missing	2 (3)
**WHO 2016**, *n* (%)		**CCSS**, *n* (%)	
MDS-SLD	5 (7.5)	Very low	1 (1.5)
MDS-MLD	20 (29.9)	Low	42 (62.7)
MDS-RS-SLD	2 (3.0)	Intermediate	11 (16.4)
MDS-RS-MLD	22 (32.8)	High	2 (3.0)
MDS-del(5q)	1 (1.5)	Very high	10 (14.9)
MDS-U	1 (1.5)	Missing	1 (1.5)
MDS-EB-1	9 (13.4)		
MDS-EB-2	7 (10.4)		
**WHO 2022** ^a^, *n* (%)		**Treatment**, *n* (%)	
MDS-del(5q)	1 (1.5)	Supportive care and growth factors	38 (56.7)
MDS-LB	26 (38.8)	Lenalidomide	6 (9.0)
MDS-LB-RS	24 (35.8)	Azacitidine	7 (10.4)
MDS-IB1	9 (13.4)	Chemotherapy	5 (7.5)
MDS-IB2	7 (10.4)	Stem cell transplantation	7 (10.4)
		Missing	4 (6.0)

Clinical characteristics of included MDS patients.

*CCSS* comprehensive cytogenetic scoring system, *IPSS-R* Revised International Prognostic Scoring System, *MDS-5q* MDS with isolated del(5q), *MDS-EB* MDS with excess blasts, *MDS-IB* MDS with increased blasts, *MDS-LB* MDS with low blasts, *MDS-LB-RS* MDS with low blasts and ≥ 15% ring sideroblasts, *MDS-MLD* MDS with multilineage dysplasia, *MDS-SLD* MDS with single lineage dysplasia, *MDS-RS-MLD* MDS with multilineage dysplasia with ring sideroblasts, *MDS-RS-SLD* MDS with single lineage dysplasia with ring sideroblasts, *MDS-U* MDS unclassifiable.

^a^The terminology of the WHO 2022 classification is used, despite the lack of molecular data since sequencing was not routinely performed at the time the study was running. In one patient (MDS40), the percentage of blasts was based on a diagnostic flow cytometry panel due to poor quality of the BM smear.

**Table 2:** Overview of the CD34^+^ cell populations.

	Manual Label	MEM
1	GMPs	FSC^+3^ SSC^+3^ CD45RA^+2^ Combi^+2^ CD33^+2^ CD123^+1^ CD38^+1^ CD44^+1^ CD45^+1^
2	CD44^dim^ CMPs	FSC^+1^ SSC^+1^ CD123^+1^ CD33^+1^ CD38^+1^ CD44^−3^ CD45RA^−2^
3	GMPs	CD45RA^+2^ CD33^+2^ Combi^+1^ CD123^+1^ CD38^+1^ CD44^+1^ CD45^+1^
4	GMPs	FSC^−2^ SSC^−^^2^ CD33^−1^ CD44^−^^1^ CD45^−1^
5	LSCs	CD45RA^+1^ CD123^+1^ CD44^+1^ CD38^−4^ CD33^−1^
6	CD44^dim^ HSCs	CD44^−4^ FSC^−3^ CD33^−3^ CD38^−3^ Combi^−2^ CD123^−2^ SSC^−1^ CD45RA^−1^ CD34^−1^ CD45^−1^
7	HSCs	CD34^+1^ CD38^−5^ CD45RA^−3^ Combi^−2^ FSC^−1^ SSC^−1^ CD123^−1^ CD33^−1^
8	SSC^high^ CD38^dim^ HSCs	SSC^+4^ FSC^−1^ CD33^−1^ CD38^−1^ CD44^−1^
9	SSC^low^ CMPs	CD33^+1^ CD45RA^−3^ SSC^−1^ Combi^−1^ CD45^−1^
10	CD34^dim^ progenitors	FSC^+4^ SSC^+4^ CD33^+2^ Combi^+1^ CD38^+1^ CD34^−3^ CD45RA^−1^ CD123^−1^
11	CMPs	FSC^+3^ SSC^+2^ CD33^+1^ CD38^+1^ CD44^+1^ CD45^+1^ CD45RA^−2^
12	MDP	FSC^+2^ SSC^+2^ CD33^+2^ Combi^+1^ CD38^+1^ CD44^+1^ CD45^+1^ CD123^−5^
13	CD45^low^ CD38^dim^ HSCs	CD123^−5^ CD45RA^−2^ FSC^−1^ SSC^−1^ Combi^−1^ CD44^−1^ CD45^−1^
14	CD38^dim^ HSCs	CD34^+1^ CD45^+1^ CD45RA^−3^ Combi^−3^ CD33^−2^ SSC^−1^ CD123^−1^ CD38^−1^
15	LSCs	SSC^+4^ Combi^+2^ CD45^+2^ FSC^+1^ CD38^−3^ CD45RA^−2^ CD34^−2^ CD123^−1^
16	CD33^−^ progenitors	FSC^+2^ SSC^+1^ CD38^+1^ CD44^+1^ CD33^−5^
17	CLPs	CD44^+^ CD33^−4^ CD123^−3^ FSC^−1^ SSC^−1^ Combi^−1^ CD44^−1^
18	CD33^low^ CMPs	CD33^−4^ CD45RA^−3^ FSC^−2^ SSC^−2^ CD45^−2^ Combi^−1^
19	CD44^−^ HSCs	CD44^−10^ FSC^−3^ Combi^−3^ CD33^−3^ CD123^−2^ CD38^−2^ SSC^−1^ CD45RA^−1^ CD34^−1^ CD45^−1^
20	CD44^dim^ HSCs	SSC^+3^ FSC^+1^ CD45^+1^ CD44^−5^ CD38^−3^ CD33^−2^ CD45RA^−1^ CD123^−1^ CD34^−1^
21	CD34^dim^ progenitors	FSC^+4^ SSC^+3^ CD33^−4^ Combi^−3^ CD34^−3^ CD123^−2^ CD38^−2^ CD45RA^−1^ CD45^−1^
22	MEPs	FSC^+3^ SSC^+2^ CD38^+1^ CD123^−4^ CD33^−3^ CD45RA^−2^ Combi^−2^
23	CD33^−^ progenitors	CD33^−6^ FSC^−1^ SSC^−1^ CD44^−1^ CD45^−1^
24	CD45^low^ HSCs	CD38^−5^ CD33^−4^ FSC^−2^ SSC^−2^ Combi^−2^ CD45^−1^
25	Lymphoid progenitors	CD38^+1^ CD33^−4^ CD44^−4^ FSC^−3^ SSC^−3^ CD123^−3^ Combi^−1^ CD45^−1^

The 25 separate CD34+ populations were labelled manually and quantitatively based on biaxial dot plots and MEM, respectively. The quantitative labels for each of the CD34^+^ populations are relative to all other populations. Note that the CD34 and CD45 expressions are relative to the pre-gated CD45dim CD34^+^ HSPC compartment.

*CLPs* common lymphoid progenitors, *CMPs* common myeloid progenitors, *GMPs* granulocyte-monocyte progenitors, *HSCs* haematopoietic stem cell, *LSCs* leukaemic stem cells, *MDP* macrophage/dendritic progenitor, *MEM* marker enrichment modelling, *MEPs* megakaryocyte-erythroid progenitors, *prog* progenitors.

### The CD34^+^ compartment in MDS demonstrates overlap with AML and benign controls

**Fig. 2:**
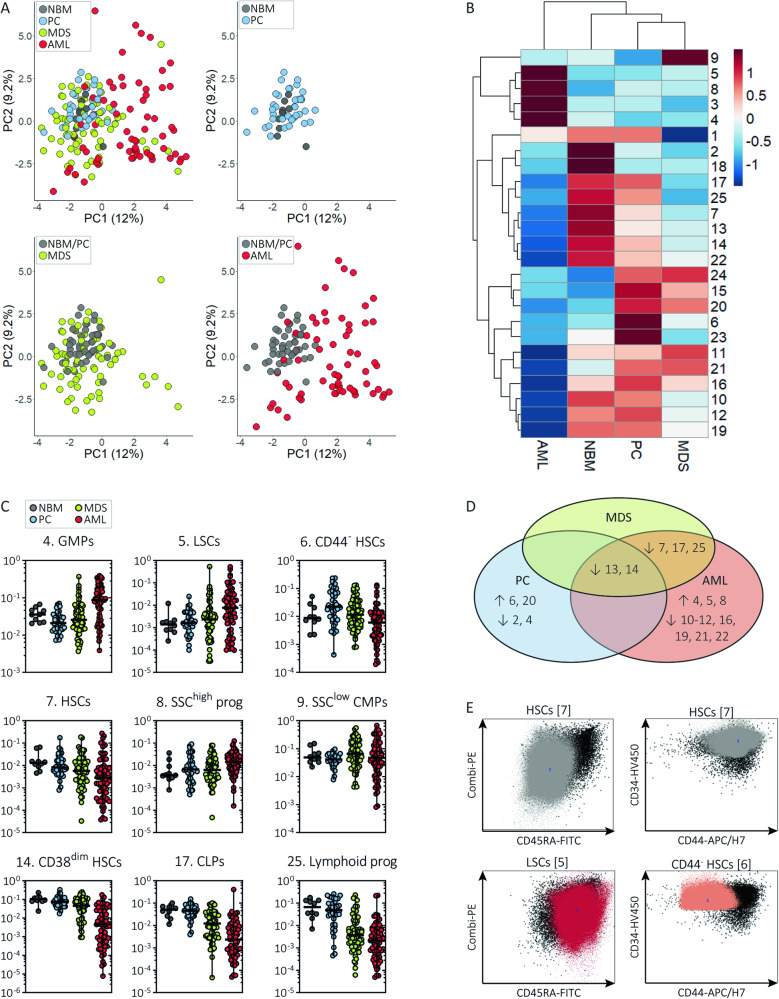
The CD34^+^ subset composition reveals no unifying feature for all MDS patients. **A** A principal component analysis applied on the CD34^+^ population frequencies for each sample. While PCs cluster with NBMs, MDS and AML samples are scattered throughout the plot indicating their variance within the CD34^+^ cell composition. In contrast to AML samples placed aside from NBMs and PCs, MDS samples overlap with controls and AML samples. The differences in the relative number of the CD34^+^ populations between diagnostic groups were tested for statistical significance using the Mann-Whitney U test (see Table S3). **B** A heatmap summary illustrating the scaled median values of the CD34^+^ population frequencies for each diagnosis. The difference in the frequency of GMPs [1] and SSC^low^ CMPs [9] between MDS and NBMs reached no statistical significance (see Table S3). **C** Dots indicating individual measures of relative frequencies of CD34^+^ populations between diagnoses. The bars indicate the median and range. Note that the Y axis has a logarithmic scale. **D** Summary of statistical differences in CD34^+^ population frequencies between MDS and related entities (adapted from Table S3). The MDS-derived CD34^+^ compartment shows decreased CD38^dim^ HSCs [13,14] and decreased HSCs [7], CLPs [17] and lymphoid progenitors [25] similar to PCs and AML samples and to AML samples, respectively. **E** Scatter plots illustrate marker expressions of two immunophenotypically abnormal stem cell populations and one normal stem cell population. Each dotplot contains cells from all samples: cells from the selected population are plotted in a colour (phenotypically normal HSCs [7] in grey, LSCs [5] in red and CD44^dim^ HSCs [6] in pink) while cells from the unselected CD34^+^ populations are plotted in black. AML acute myeloid leukaemia, CLPs common lymphoid cells, CMPs common myeloid progenitors, GMPs granulocyte-monocyte progenitors, HSCs haematopoietic stem cells, LSCs leukaemic stem cells, MDS myelodysplastic neoplasms, NBM normal bone marrow, PC pathological controls.

Since MDS can be challenging to diagnose due to related disorders, we aimed to identify cell populations discriminating between MDS, AML and controls. Compared with NBMs, the MDS-derived MNC compartment contained relatively increased percentages of CD34^-^ and CD34^+^ progenitors [II,IV] (*P* = 0.005, *P* = 0.049) and erythroid cells [X] (*P* = 0.002) next to decreased percentages of lymphocytes [IX] (*P* = 0.006; Fig. S6A). Similarly, AML patients showed increased CD34^−^ progenitors [II] (*P* = 0.025), CD34^+^ progenitors [IV,VIII] (*P* < 0.001, *P* = 0.004) and lymphocytes [IX] (*P* < 0.001), whereas PCs had increased SSC^low^ granulocytes [III] (*P* = 0.006) and erythroid cells [X] (*P* < 0.001) but comparable progenitor percentages. Next, we focused on the CD34^+^ compartment and labelled the FlowSOM tree by diagnosis. We observed no CD34^+^ populations that were uniquely present in MDS (Fig. S4). Second, we applied a principal component analysis on the CD34^+^ population abundancies for each of the samples individually (Fig. 2A). This analysis clustered NBMs with PCs, grouped AML aside from controls but scattered MDS samples, indicating a heterogeneous CD34^+^ cell composition. Third, we compared CD34^+^ population abundancies between subjects (statistics in Table S3). Compared with NBMs, MDS patients showed reduced percentages of HSCs [7], CD38^dim^ HSCs [13,14], CLPs [17] and lymphoid progenitors [25] (Fig. 2B, C). However, none of these perturbations were specific for MDS (Fig. 2D). Also, AML patients had decreased HSCs [7], CD38^dim^ HSCs [13,14], CLPs [17] and lymphoid progenitors [25], whereas PCs had reduced CD38^dim^ HSCs [13,14]. Contrarily, we observed a distinctive HSPC profile for AML, including increased percentages of LSCs [5], SSC^high^ CD38^dim^ HSCs [8] and GMPs [4] at the expense of other CD34^+^ populations. The LSCs [5] were predominantly characterised by the expression of CD45RA (Fig. 2E). Different from AML, PCs demonstrated decreased percentages of GMPs [4] beside increased CD44^dim^ HSCs [6,20]. Interestingly, a separate analysis of stem cell populations showed moderately increased LSCs [5] and CD44^dim^ HSCs [6] at the expense of normal HSCs [7] in MDS patients, suggesting overlap with both AML and PCs, respectively (Fig. S6B). In short, AML patients have an unique HSPC signature that consists of LSCs and expanded GMPs, whereas the HSPC compartment from MDS patients is more heterogeneous and not distinctive for the diagnosis.

### The CD34^+^ cell composition captures clinical and prognostic value in MDS

**Fig. 3:**
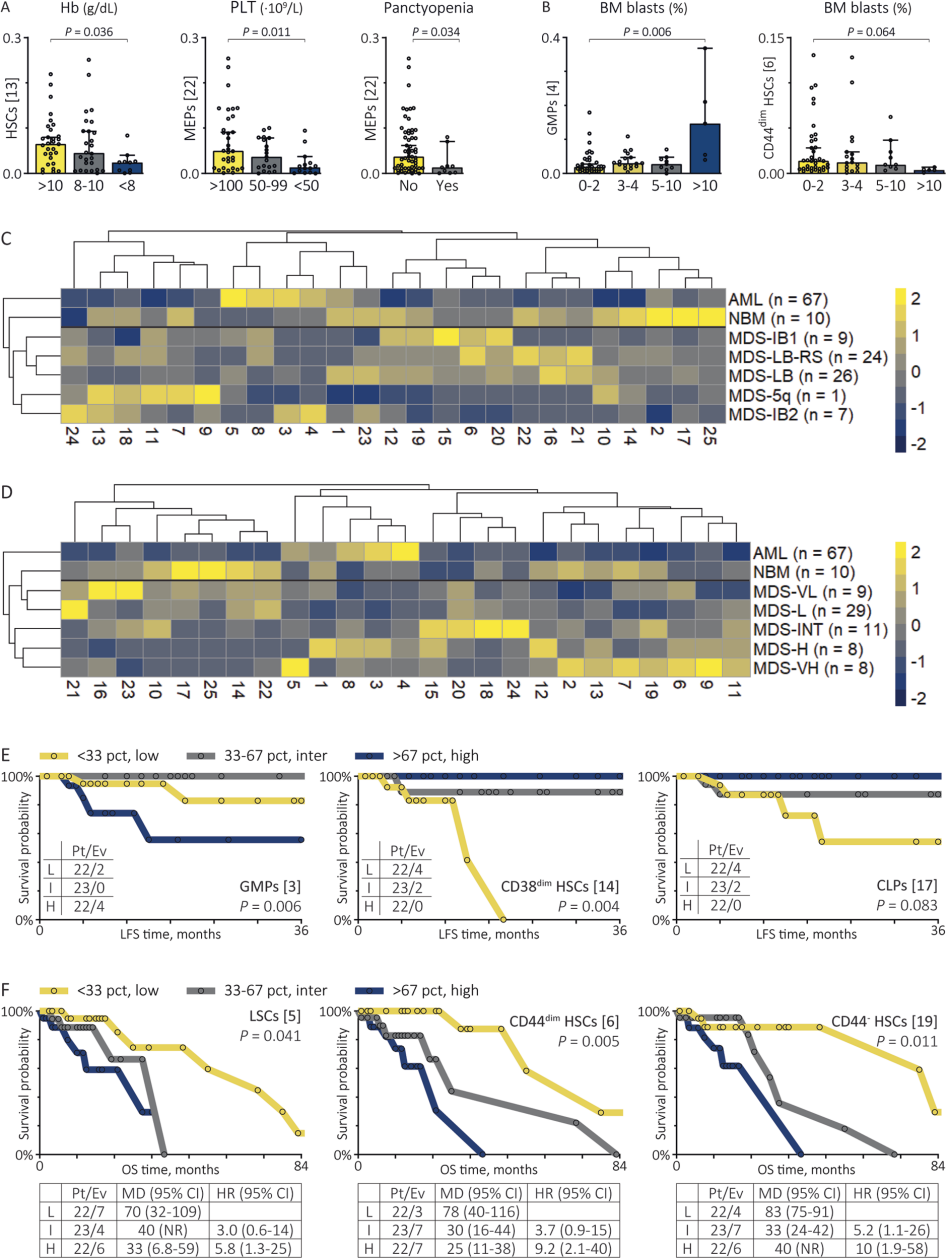
The heterogeneous CD34^+^ cell composition of MDS patients has clinical significance. **A, B** Examples of the relationships between CD34^+^ populations and PB and BM blast counts (see Table 3). Median values with the range are shown. *P* values are derived from the Kruskal-Wallis and Mann-Whitney U tests. **C, D** Heatmap summary of the median CD34^+^ population percentages in MDS patients next to NBMs and AML patients as reference groups. The Mann-Witney U test was applied on the CD34^+^ population percentages from MDS patients stratified into one category compared with all MDS patients from other WHO and IPSS-R categories. Amongst others, MDS-LB-RS patients had increased CD44^dim^ HSCs [6] (*P* = 0.009) and MEPs [22] (*P* < 0.001), whereas MDS-IB2 patients had decreased MEPs [22] (*P* = 0.001), CD44^−^^/dim^ HSCs [6,19,20] (*P* = 0.003, *P* = 0.006, *P* = 0.001) and CD44^dim^ CMPs [2] (*P* = 0.021). Moreover, MDS-VL patients had increased CLPs [17] (*P* = 0.038) and CD33^-^ progenitors [16,23], MDS-L patients had increased MEPs [22] (*P* = 0.040) and reduced GMPs [1,3] (*P* = 0.029, *P* = 0.019) and MDS-VH patients had and increased LSCs [5] (*P* = 0.005) and SSC^low^ CMPs [9] (*P* = 0.010). **E, F** Kaplan-Meier curves with the log-rank test illustrating the prognostic value of CD34^+^ populations stratified into three groups based on the 33rd and 67th percentiles in MDS patients for leukaemic transformation and all-cause mortality. Six examples are shown (Table 3). The tables below the overall survival curves summarise the patients/events-ratio, median survival times and hazard ratios with the 95% confidence interval. The tables within the leukaemia-free survival curves summarise patients/events-ratio only since the median survival times were not reached. Patients undergoing induction chemotherapy or stem cell transplantation were censored at the date of treatment start. HSCs haematopoietic stem cells, LSCs leukaemic stem cells, GMPs granulocyte-monocyte progenitors, MDS-LB-RS MDS with low blasts and ring sideroblasts, MEPs megakaryocyte-erythroid progenitors, CLPs common lymphoid cells, VL very low risk, L low risk, INT intermediate risk, H high risk, VH very high risk, MDS-5q MDS with isolated del(5q), MDS-LB MDS with low blasts, MDS-IB MDS with increased blasts, OS overall survival, LFS leukaemia-free survival, Pt/Ev patients/events, MD median survival times, HR hazard ratio, CI confidence interval.

Further analysis focused on MDS to dissect their clinical heterogeneity based on immunophenotypes. First, we studied the relationships of MNC populations between clinical outcomes, PB values and blast counts. Contrary to the FSC^high^ CD34^+^ population [IV], the FSC^dim^ CD34^+^ population [VIII] related to unfavourable features, including a shorter LFS (*P* = 0.033) and EFS (*P* = 0.002) as well as reduced platelets (*P* = 0.018) and increased BM blasts (*P* = 0.004), both tested as categorical variables adopting IPSS-R cut-offs (Fig. S7). From the remaining MNC populations, we only observed a trend between reduced lymphocytes [I] and expanded PB and BM blasts (*P* = 0.052, *P* = 0.093). Second, we studied the relationships of CD34^+^ populations between (I) PB values and blast counts, (II) clinical scores and (III) patient outcomes. From the CD34^+^ populations, 10/25 and 14/25 populations related to favourable and unfavourable clinical characteristics, respectively (statistics summarised in Table 3). (I) The CD38^dim^ HSCs [14], CLPs [17] and MEPs [25] correlated with higher PB values and lower BM blasts (Fig. 3A, B). Increased LSCs [15] and GMPs [3,4] were related to expanded BM blasts, whereas BM and PB blasts correlated inversely with CD44^−^^/dim^ HSCs [6,19,20] and CD44^dim^ HSCs [6], respectively. (II) Next, we studied the CD34^+^ compartment in MDS patients stratified by the WHO and IPSS-R. In general, we observed increased lymphoid populations and MEPs in lower-risk IPSS-R categories and increased LSCs, CD44^−^ HSCs and GMPs in higher-risk IPSS-R categories. Amongst others, MDS patients with low blasts and ring sideroblasts had expanded CD44^dim^ HSCs [6,20] than other WHO categories, whereas MDS-IB2 patients had decreased CD44^−^^/dim^ HSCs [6,19,20] and decreased CD44^dim^ CMPs [2] (statistics summarised in legend Fig. 3). (III) Third, we studied the prognostic value of CD34^+^ population percentages re-coded as categorical variables using the 33th and 67th percentiles (Table 3). High numbers of CD38^dim^ HSCs [14], CLPs [17] and MEP [22] correlated with a longer LFS and EFS (Fig. 3E). In contrast, expansion of GMPs [3,4] increased the risk of leukaemic transformation and disease progression and high numbers of LSCs [5] and CD45^low^ HSCs [24] increased the risk of all-cause mortality (Fig. 3F). An expansion of CD44^dim/−^ HSCs [6,19] related also to a short survival time, despite their inverse relationship with blast counts. Although reaching no statistical significance, MDS patients with leukaemic transformation appeared to have increased LSCs [5] compared with patients with stable disease (Fig. S7). These data confirm the clinical significance of immunophenotypically aberrant stem cells, the expansion of GMPs and the reduction of MEPs and CLPs [20]. Moreover, our results indicate that downregulated CD44 expression on stem cells is associated with a poor clinical outcome in cases without accompanying increases in blast counts.

**Table 3:** Statistical summary of the clinical value of CD34^+^ cell populations.

		PanC	Hb^a^	PLT^a^	BM-BL^a^	PB-BL^c^	IPSS-R	CCSS	Ost^b^	LFSt^b^	EFSt^b^
		yes/no	g/dL	∙10^9^/L	%	%			months	months	months
**Favourable**											
10	CD34^dim^ prog	0.031^−^	ns	ns	ns	ns	ns	ns	ns	0.061^+^	0.076^+^
14	CD38^dim^ HSCs	0.012^−^	0.036^+^	ns	ns	ns	0.043^−^	ns	0.001^+^	0.004^+^	<0.001^+^
16	CD33^−^ prog	ns	ns	ns	ns	ns	0.033^−^	ns	ns	ns	ns
17	CLPs	0.055^−^	ns	0.025^+^	ns	ns	0.022^−^	ns	ns	0.083^+^	0.094^+^
18	CD33^low^ CMPs	ns	0.050^+^	ns	ns	0.011^d^	ns	ns	ns	ns	ns
20	CD44^dim^ HSCs	ns	ns	ns	0.007^−^	ns	ns	ns	ns	ns	ns
21	CD34^dim^ prog	0.015^−^	ns	0.048^+^	<0.001^−^	0.013^−^	0.005^−^	ns	ns	ns	ns
22	MEPs	0.034^−^	ns	0.011^+^	0.001^−^	0.063^−^	0.060^−^	ns	ns	ns	0.051^+^
23	CD33^−^ prog	ns	ns	ns	ns	ns	0.024^−^	ns	ns	ns	ns
25	Lymphoid prog	ns	ns	0.016^+^	ns	ns	0.072^−^	ns	ns	0.095^+^	ns
**Unfavourable**											
1	GMPs	0.051^+^	ns	0.082^−^	ns	ns	ns	ns	ns	ns	ns
2	CD44^dim^ CMPs	ns	0.038^−^	ns	ns	ns	ns	0.038^+^	ns	ns	ns
3	GMPs	ns	ns	ns	0.028^+^	ns	ns	ns	ns	0.006^−^	<0.001^−^
4	GMPs	ns	ns	ns	0.006^+^	0.027^+^	ns	0.061^+^	ns	0.070^−^	0.003^−^
5	LSCs	ns	ns	ns	ns	0.065^d^	0.001^+^	0.012^+^	0.041^−^	ns	ns
6	CD44^dim^ HSCs	ns	ns	ns	0.064^−^	0.069^−^	ns	ns	0.005^−^	ns	0.061^−^
7	HSCs	ns	ns	ns	ns	0.092^d^	ns	0.094^+^	ns	ns	ns
8	SSC^high^ CD38^dim^ HSCs	ns	0.017^−^	ns	ns	ns	ns	0.086^+^	ns	ns	ns
9	SSC^low^ CMPs	ns	ns	0.067^−^	ns	ns	0.007^+^	0.037^+^	ns	ns	ns
11	CMPs	ns	ns	ns	ns	ns	ns	0.015^+^	ns	ns	ns
13	CD45^low^ CD38^dim^ HSCs	ns	ns	ns	ns	ns	0.021^+^	ns	ns	ns	ns
15	LSCs	ns	ns	ns	0.026^+^	0.086^d^	ns	ns	ns	ns	ns
19	CD44^−^ HSCs	ns	ns	ns	0.076^−^	ns	ns	0.073^+^	0.011^−^	ns	0.036^−^
24	CD45^low^ HSCs	ns	ns	ns	ns	0.085^+^	0.079^+^	ns	0.032^−^	ns	ns

*P*-values represent statistical comparisons between CD34^+^ populations frequencies and clinical features based on the Mann-Whitney U and Kruskal-Wallis tests. Favourable populations correlated positively with PB values and survival times, but inversely with blast counts and risk scores. Apart from CD44^dim/^^−^ HSCs [6,19] showing a trend towards lower blast percentages, unfavourable populations correlated positively with blast counts and risk scores, but inversely with PB values and survival times. We observed no significant relationships between MDP [12] and clinical features nor between CD34+ populations and neutrophil counts.

*BM-BL* bone marrow blasts, *CCSS* comprehensive cytogenetic scoring system, *CLPs* common lymphoid progenitors, *CMPs* common myeloid progenitors, *EFS* event-free survival, *GMPs* granulocyte-monocyte progenitors, *Hb* haemoglobin, *HSCs* haematopoietic stem cell, *LFS* leukaemia-free survival, *LSCs* leukaemic stem cells, *MEPs* megakaryocyte-erythroid progenitors, *MDP* macrophage/dendritic progenitors, OS overall survival, *PanC* pancytopenia, *PB-BL* peripheral blood blasts, *PLT* platelets, *prog* progenitors.

^a^Stratified into groups following the IPSS-R.

^b^Value of CD34^+^ populations stratified into groups based on the 33rd and 67th percentiles for predicting OS, LFS and EFS.

^c^Stratified into 0–1%, 2–4% and 5–19% PB-BM blasts. +/− Positive/Negative relationship.

^d^Non-linear relationship.

### The CD34^+^ subset composition changes during the disease course

**Fig. 4:**
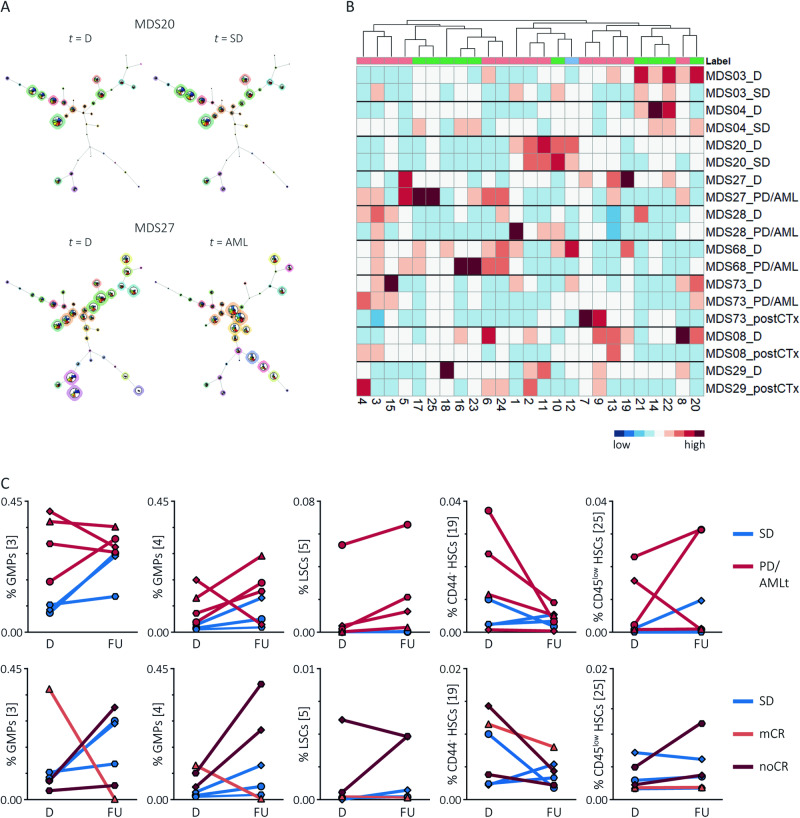
Sequential sampling during leukaemic evolution demonstrates outgrowth of unfavourable CD34^+^ populations. See also Fig. S8. Samples at time of diagnosis (*n* = 9) were compared with sequential samples during follow-up (*n* = 10). From one patient (MDS73), samples were collected during disease progression and after stem cell transplantation. **A** FlowSOM trees illustrating the CD34^+^ composition at diagnosis and follow-up. Two examples are shown. Patient MDS20, diagnosed with MDS-LB, had a stable disease 6 months after diagnosis and an unchanged CD34^+^ composition. Patient MDS27, diagnosed with MDS-LB-RS, progressed to leukaemia 6 months after diagnosis and showed, amongst others, expanded LSCs [5], CD45^low^ HSCs [13] and GMPs [3,4] at the expanse of HSCs [7], CD44^−^ HSCs [19], CMPs [2,9,11] and MEPs [22] (see Fig. 4C). **B** Heatmap summary of the median percentages of CD34^+^ progenitors at diagnosis and follow-up in MDS patients with a stable disease, disease progression or leukaemic transformation and a residual disease (MDS08, MDS29) or morphological complete remission (MDS73) after cytotoxic therapy. The colour annotation of the CD34^+^ populations indicate the manual labels as summarised in Table 3, i.e., favourable (green), unfavourable (red) and unknown (blue, only population 12) **C** Correlation figures illustrating the change in several CD34^+^ progenitor percentages at the time of diagnosis and follow-up in MDS patients with disease progression or leukaemic transformation (upper panel) and a residual disease or morphological complete remission after chemotherapy (lower panel). In each figure, the percentages from MDS patients with a stable disease are illustrated as comparison. Abbreviations: CMPs, common myeloid progenitors; GMPs, granulocyte-monocyte progenitors; HSCs, haematopoietic stem cells; LSCs leukaemic stem cells; mCR, morphological complete remission; MEPs, megakaryocyte-erythroid progenitors; MDS-LB, MDS with low blasts; MDS-LB-RS, MDS with low blasts and ring sideroblasts; noCR, no complete remission; PC, principal components; PD/AML, progressive disease or leukaemic transformation; postCTx, after cytotoxic therapy (chemotherapy or allogeneic stem cell transplantation); PD stable disease, *t* time.

In search of the haematopoietic populations that are involved in leukaemogenesis, we selected MDS patients with sequential BM samples and mapped the pre-processed fcs files to the FlowSOM trees (examples in Fig. 4A). Considering the small sample size, we used descriptive analyses to compare MDS patients with progressive disease or leukaemic transformation (PD/AMLt, *n* = 4), MDS patients with residual disease or morphological complete remission after chemotherapy or stem cell transplantation (post-CTx, *n* = 3) and MDS patients with a stable disease (SD, *n* = 3) following treatment with growth factors, lenalidomide or azacitidine (Table S4). The MNC compartment from PD/AMLt patients contained expanded CD34^+^ progenitors [VIII] at the time of disease evolution, whereas the MNC compartment from SD patients was relatively maintained (Fig. S8A). To explore the variance within the CD34^+^ compartment during follow-up, we applied a principal component analysis on the CD34^+^ population abundancies (Fig. S8B). Only the CD34^+^ subset composition from SD patient MDS20 remained stable during the disease course. In contrast, the diagnosis and follow-up samples from the remaining SD patients, PD/AMLt and post-CTx patients did not cluster, indicating an altered CD34^+^ composition over time (Fig. 4B). Despite the heterogeneity within the CD34^+^ composition between both patients and time points, there might be some trends in CD34^+^ cell population kinetics between patient groups. In most PD/AMLt patients, we observed an increase in LSCs [5] and GMPs [4] and a decrease in CD44^−^ HSC [19] at the time of disease evolution (Fig. 4C). Moreover, the post-treatment samples from patients with residual disease (MDS08 and MDS29) contained expanded LSCs [5] and GMPs [4], whereas the post-CTx patient with morphological complete remission (i.e. less than 5% blasts, MDS73) had expanded HSCs [7] and SSC^low^ CMPs [9] at the expense of, amongst others, GMPs [4]. Despite the limitations of this sequential BM analysis, including the low number of samples and distinct treatment modalities, these observations may suggest that disease progression and relapse in MDS is associated with an expansion of LSCs and GMPs, but not with CD44^−^ HSCs.

### Dissection of MDS into clinically relevant subtypes based on CD34^+^ cells: a new approach

**Fig. 5:**
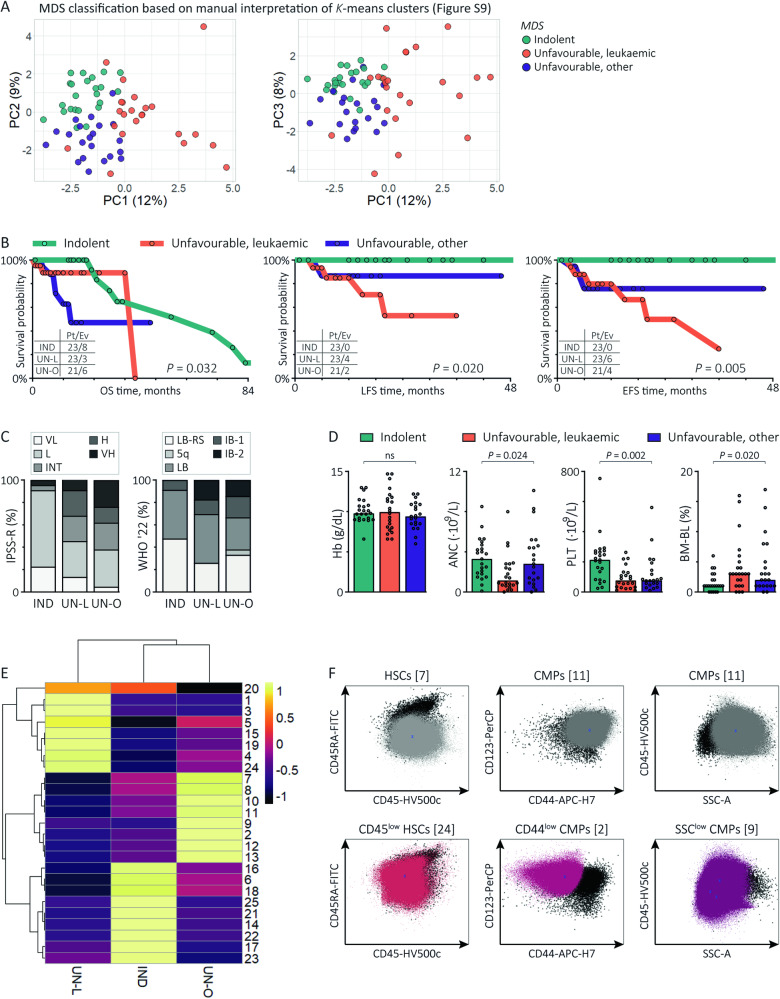
MDS classified into clinically relevant subtypes based on the CD34^+^ subset composition. **A** MDS patients classified into 3 subtypes based on *K*-means clustering (*k* = 9) applied on the 26 principal components of the FlowSOM populations and manual interpretation of 9 *K*-means clusters using the prevalence of AML and NBM samples within each cluster as reference as shown in Fig. S9 and Table S5. **B** Kaplan-Meier curves with the log-rank test illustrating the prognostic value of the MDS subtypes for all-cause mortality and leukaemic transformation. **C** Stacked histograms illustrating the distribution of the IPSS-R risk scores and WHO 2022 categories for each MDS subtype. **D** Graph bars illustrating the median values of PB values and BM blast counts for each MDS subtype. *P* values are derived from the Kruskal-Wallis test. **E** Heatmap summary of the median CD34^+^ population percentages for each MDS subtype. ANC absolute neutrophil count, BM-BL bone marrow blasts, CMPs common myeloid progenitors, H IPSS-R high-risk, Hb haemoglobin, HSCs haematopoietic stem cells, IND indolent subtype, INT IPSS-R intermediate-risk, L IPSS-R low-risk, LFS leukaemia-free survival, MDS-5q MDS with isolated del(5q), MDS-IB MDS with increased blasts, MDS-LB MDS with low blasts, MDS-LB-RS MDS with low blasts and ring sideroblasts, OS overall survival, PC principal component, PLT platelets, UN-L unfavourable, leukaemic subtype, UN-O unfavourable, other subtype, VH IPSS-R very high-risk, VL IPSS-R very low-risk.

Finally, we investigated the possibility to distinguish MDS into indolent and more aggressive subtypes. We hypothesised that MDS samples resembling AML or NBM based on the CD34^+^ subset composition reflect leukaemic and indolent subtypes, respectively, whereas MDS resembling neither AML nor NBM might indicate an unfavourable non-leukaemic subtype. However, our data did not support a 3-class split of MDS. Therefore, we manually labelled MDS patients by the aid of computational clustering to present an alternative method of classifying MDS. We over-clustered the principal components of the CD34^+^ population frequencies from the 183 samples using *K*-means clustering with a manually chosen number of clusters (*k* = 9). Subsequently, we manually classified the nine clusters into three groups or subtypes based on the proportion of MDS, AML and NBM samples (Fig. 5A, Fig. S9 and Table S5). We discriminated an indolent (*n* = 23, 34.3%), a leukaemic (*n* = 23, 34.3%) and a third subtype (*n* = 21, 31.3%). Compared with the other patients, MDS patients from the leukaemic subtype showed an increased risk of leukaemic transformation (*P* = 0.018) and disease progression (*P* = 0.014) but a comparable risk of all-cause mortality (*P* = 0.997; Fig. 5B). Differently, MDS patients from the third subtype had an shorter survival time (*P* = 0.012) in comparison with indolent and leukaemic MDS, whereas the difference in the risk of leukaemic transformation (*P* = 0.536) and disease progression (*P* = 0.250) was not statically significant. The dissimilar clinical outcome of the three subtypes was reflected by their risk distribution following the IPSS-R (*P* = 0.022), with more very low-risk and low-risk patients into the indolent subtype and more intermediate-risk to very high-risk patients into the leukaemic and third subtype (Fig. 5C). Moreover, MDS patients from the indolent subtype had lower BM blasts (*P* = 0.006), higher platelets (*P* < 0.001) and a trend towards higher neutrophils (*P* = 0.060) compared with other subtypes (Fig. 5D). Vice versa, MDS patients from the leukaemic subtype had decreased platelets (*P* = 0.024) and neutrophils (*P* = 0.008) next to a trend towards increased BM blasts (*P* = 0.072). Finally, we questioned which CD34^+^ populations contributed to the discrimination between subtypes (Fig. 5E). Indolent MDS patients showed the highest percentages of CD38^dim^ HSCs [14], CLPs [17], lymphoid progenitors [25], CD33^−^ progenitors [16], MEPs [22] and CD34^dim^ progenitors [21] next to lowest percentages of LSCs [5], CD45^low^ HSCs [24], GMPs [4] and SSC^low^ CMPs [9], suggesting some preservation of normal haematopoiesis (statistics in Table S6). Resembling AML, leukaemic subtype MDS patients showed increased percentages of LSCs [5], CD45^low^ HSCs [24] and GMPs [1,3,4] at the expense of CD38^dim^ HSCs [13,14], CLPs [17], lymphoid progenitors [25], CMPs [2,11], MEPs [22] and CD34^dim^ progenitors [21]. Third subtype patients also showed reduced percentages of CLPs [17] and lymphoid progenitors [25], but differed from the leukaemic subtype by decreased GMPs [3] and expanded CD45^low^ CD38^dim^ HSCs [13], MDP [12] and various CMP subsets [2,9,11] that partly expressed aberrant immunophenotypes, including CD44^dim^ CMPs [2] and SSC^low^ CMPs [9] (Fig. 5F). Despite our low sample size, these data demonstrate the feasibility of dissecting MDS into an indolent subtype, a leukaemic subtype and another unfavourable subtype based on the CD34^+^ composition. Our data suggest that indolent MDS maintain normal haematopoietic progenitors, whereas leukaemic MDS and other unfavourable MDS are characterised by disruption of normal haematopoiesis and the expansion of LSCs and CMPs, respectively. Nonetheless, considering the difficulty of this method and the lack of validation and robustness, this methodology is not more than an example.

## Discussion

A major focus in translational MDS research is the development of more informative classifications. Recently, insights into the molecular pathogenesis of MDS led to the introduction of revised classifications, including the WHO 2022, ICC and IPSS-M [14–16]. However, the mutational landscape of MDS is heterogeneous and little progress has been made with respect to the discrimination of distinct clinical outcomes. In AML, the complementary value of detecting LAIPs by flow cytometry next to molecular profiling has been demonstrated [19]. By applying computational clustering to immunophenotypes of CD34^+^ cells in 183 patients and controls, this study revealed heterogeneity within the MDS-derived CD34^+^ compartment. We linked several CD34^+^ populations to clinical features and outcomes in MDS patients and demonstrated the feasibility of dissecting MDS into an indolent, leukaemic and another unfavourable subtype based on the CD34^+^ composition.

Since the diagnosis of MDS can be challenging, several tools have been developed that help diagnose MDS [23–26]. In a comparison of distinct flow cytometry (FCM) scores, the integrated MDS-FCM score achieved the highest diagnostic accuracy for separating MDS from non-clonal cytopenic disorders [24, 27]. Similarly to the MDS-FCM score, the Ogata FCM score and the computational approach from Duetz et al. achieved high diagnostic power but were trained to separate low-risk MDS from non-clonal cytopenic disorders [23, 25]. One of our objectives was to identify a CD34^+^ signature specific for MDS. However, reflecting MDS’ heterogeneous nature, MDS patients demonstrated a highly heterogeneous CD34^+^ composition with aberrancies, including reduced HSCs and lymphoid progenitors, that were also observed in some AML patients and controls. By exposing the variance within the CD34^+^ composition, these data indicate the difficulty of identifying an immunophenotypic biomarker for MDS as one entity.

The value of the LSC frequency in AML is widely accepted for predicting survival and relapse at diagnosis and post-treatment, respectively [28, 29]. In MDS, the myeloid progenitor count is used as a prognostic parameter in FCM scores, whereas we recently showed that manually gated LSCs predict leukaemic progression [20, 23, 30]. Yet, manual gating of the low-frequent LSCs is sensitive to errors. In this study, we used the same MDS dataset and demonstrated that also by computational identification of LSCs, this population has prognostic implications. Contrary to manually gated LSCs, computationally identified LSCs did not predict leukaemic transformation. This may be explained by the separation of LSCs in distinct populations based on marker expressions, the exclusion of the aberrant markers from the Combi channel and the use of percentages rather than the presence or absence of LSCs. Apart from LSCs, our results confirmed the negative impact of expanded CD34^+^ progenitors and GMPs in MDS. While the expansion of GMPs is described to characterise high-risk MDS, our results demonstrated that increased GMP percentages predict leukaemic transformation [20, 31, 32]. This observation is consistent with previous reports showing that MDS-derived GMPs upregulate CD47, leading to evasion of phagocytosis, and more frequently carry STAT2 mutations and other gene signatures involved in self-renewal, thereby driving leukaemogenesis [32–34]. Moreover, the existence of a GMP-like population with LSC activity has been suggested in AML [35, 36]. These findings make us question whether LSC and GMP frequencies hold prognostic value beyond the molecular profile in MDS and warrant future studies.

Causes of death are diverse in MDS and only a minority of patients transform to AML. However, prognostic models use all-cause mortality for estimating OS and a composite endpoint consisting of leukaemic transformation and all-cause mortality for LFS [13, 14]. Our data suggest the possibility to dissect MDS into (a) an indolent subtype, (b) a leukaemic subtype and (c) another unfavourable subtype based on (a) preservation of CLPs and MEPs, (b) expansion of LSCs and GMPs and (c) proliferation of HSCs and CMPs, respectively. It is well-known that progenitor B cells are declined in low-risk MDS and that their preservation correlates with a better prognosis [37, 38]. Our study confirmed the clinical value of CLPs. Moreover, our data revealed that the number of MEPs is inversely related to the blast count. Contrary to previous reports that associated a relative increase in CMPs with low risk MDS, we showed that MDS patients characterised by proliferated HSCs and CMP, indicative of a differentiation block at this level, have poor overall survival without leukaemic development. Previous studies described reduced and increased CD44 expression in low-risk and high-risk MDS, respectively [39, 40]. We showed that HSCs subsets with downregulated CD44 are associated with cytopenia and a shortened OS despite normal blast percentages. Moreover, our data indicate that MDS with low blasts and ring sideroblasts contain relatively high percentages of HSC subsets with downregulated CD44 expression, which is in line with the low risk of leukaemic transformation and the profound cytopenia generally found in MDS-*SF3B1*. Considering that CD44 mediates HSPC binding to the extracellular matrix and thereby regulates haematopoiesis, we hypothesise that downregulation of CD44 on HSCs contributes to impaired haematopoietic differentiation and cytopenia in MDS [41, 42]. The role of CD44 in MDS pathogenesis deserves further investigation, e.g., via genetic profiling in combination with clonogenic assays and xenotransplantation experiments of CD44^+^ versus CD44^−^^/low^ HSCs.

To conclude, we identified immunophenotypic CD34^+^ signatures that match with MDS patients’ clinical phenotype and outcome. Considering the genetic diversity in MDS, immunophenotypic analyses of CD34^+^ cells might yield prognostic information additional to the molecular status. Moreover, in the era of precision medicine, we advocate for the distinction between different clinical endpoints. The presented dissection of MDS into an indolent, a leukaemic and another unfavourable subtype should serve as an example and requires further research that tests our findings in an independent patient cohort with long-term follow-up including adjustments of multiple testing. Our exploratory work may serve as a pilot for future studies that use unsupervised analysis of CD34^+^ cells in MDS patients stratified by death cause in combination with functional and molecular analysis of the identified populations of interest.

### Supplementary information

Supplemental material

## Data Availability

The data that support the findings of this study are available upon reasonable request from the corresponding author, A. A. van de Loosdrecht.
