# Supplementary material for: Myelodysplastic neoplasms dissected into indolent, leukaemic and unfavourable subtypes by computational clustering of haematopoietic stem and progenitor cells
Source: Leukemia. 2024 Mar 8;38(6):1365–77. doi: 10.1038/s41375-024-02203-z (PMC11147773; doi:10.1038/s41375-024-02203-z)
Supplement: Supplementary file 1 — Supplemental material [file 41375_2024_2203_MOESM1_ESM.pdf]

Myelodysplastic neoplasms dissected into indolent, leukaemic and unfavourable subtypes by computational clustering of haematopoietic stem and progenitor cells

Supplementary Information

Table of Contents

Figure S1. Flow chart of the study population page 3

Table S1. Clinical characteristics of study subjects page 3

Sample preparation page 3

Flow cytometry page 4

Table 2. Leukaemic stem cell tube page 4

Figure S2. Pre-gating strategy page 5

Data pre-processing page 5

Figure S3. MNC and CD34$^+$ cell counts page 6

Clustering analysis page 6

Figure S4. Cell clustering by potential batch effects page 7

Cell cluster interpretation page 7

Figure S5. FlowSOM analysis of the MNC compartment page 8

Figure S6. Distribution of MNC and HSC populations over diagnostic groups page 9

Table S3. Statistical summary of the diagnostic value of CD34$^+$ populations page 10

Figure S7. Clinical and prognostic value of CD34$^+$ progenitors and LSCs page 11

Table S4. Overview of MDS patients with sequential samples page 12

Figure S8. MNC and CD34$^+$ cell compartment changes during follow-up page 13

Figure S9. *K*-means clustering on the principal components of the population abundancies page 14

Table S5. MDS classification based on *K*-means clustering page 15

Table S6. Statistical summary of the differences in CD34$^+$ populations between MDS subtypes page 15

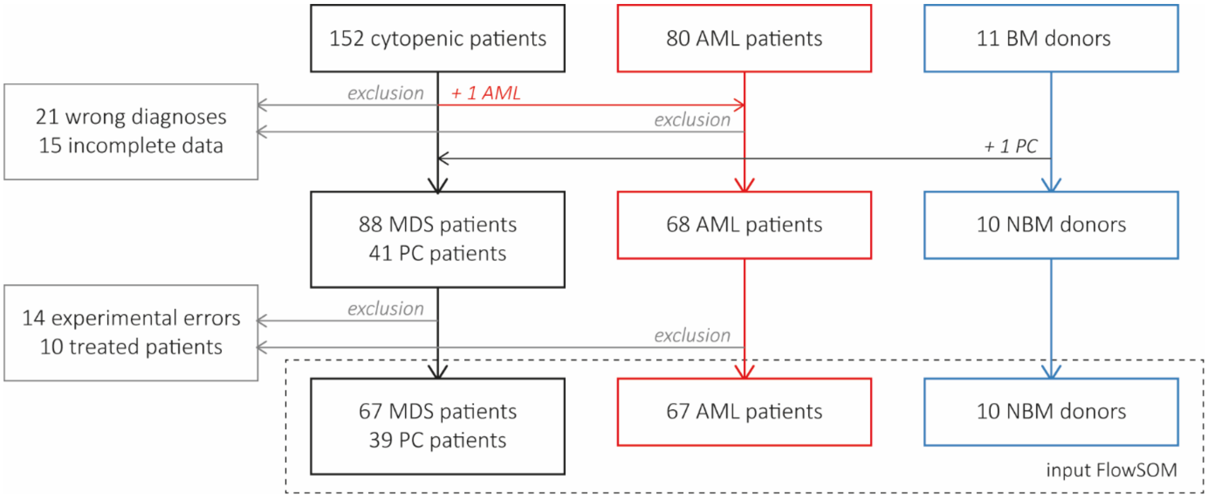

Figure S1. Flowchart of the study population

Normal bone marrow (NBM) was collected from cardiothoracic surgery patients. Patients with reactive conditions, nutritional deficiencies or non-myeloid clonal disorders were considered as pathological controls (PCs). The PC cohort contained one patient with a monoclonal gammopathy of unknown significance and three patients with a monoclonal B cell lymphocytosis (MBL). Samples were excluded in case of incomplete data, experimental errors, treatment before specimen collection and wrong diagnoses that were not of interest for this study, i.e. inconclusive cases (n = 10), CMML (n = 5), myelofibrosis (n = 2), multiple myeloma (MM, n = 1), acute erythroid leukaemia (n = 1), polycythaemia vera (n = 1) and Diamond-Blackfan anaemia (n = 1). One NBM donor was considered a PC because of the presence of MBL. Two patients with MDS in addition to MM with 10% and 12% plasma cells within the BM aspirate were not excluded.

Table S1. Clinical characteristics of study subjects

	MDS	AML	PC	NBM	*P* value
Number, n (%)	67 (36)	67 (36.6)	39 (21.3)	10 (5.5)	
Sex, n (%)					0.034
Female	16 (23.9)	31 (46.3)	17 (43.6)	4 (40)	
Male	51 (76.1)	35 (52.2)	21 (53.8)	6 (60)	
Missing		1 (1.5)	1 (2.6)		
Age, median (range)	70 (30 - 89)	59 (22 - 89)	70 (29 - 89)	68 (62 - 80)	<0.001

The study population comprised 183 patients and controls. There was a significant difference in the number of sexes and the median age at diagnosis between diagnostic groups, with the highest male predominance among MDS patients and the lowest age among AML patients. The median age at diagnosis between MDS, NBM and PC did not differ significantly. The differences in age and number of sexes between groups was tested for statistical significance using the Kruskal-Wallis and Chi-Square tests, respectively.

Sample preparation

Samples from AML patients and controls were analysed fresh. Samples from MDS patients were analysed both fresh (70%) or after cryopreservation (30%). Fresh samples were deprived of erythrocytes using an ammonium chloride-based lysing solution (PharmLyse, BD Biosciences) for 10 minutes at room temperature. Lysed samples were washed twice and re-suspended in the washing solution (phosphate

buffer saline (PBS) with 0.1% human serum albumin (HSA) and 0.05% sodium azide). Mononuclear cells (MNCs) were obtained using a Ficoll gradient (1.077 g/mL, Amersham Biosciences) and subsequent erythrocyte lysis using home-made ammonium chloride-buffered lysing solution and then cryopreserved in RPMI-1640 (Gibco, Life technologies) with 20% heat-inactivated fetal bovine serum (FBS, Greiner) and 10% DMSO (Riedel-de Haën). Cryopreserved MNCs were thawed in a 37°C water bath and recovered in preheated RPMI with 20% FBS, 0.1% DNAse (Sigma-Aldrich) and 0.1% $MgCl_2$ [1M]. Thawed MNCs were washed and re-suspended in PBS with 0.1% HSA.

Flow cytometry

Prepared cells were incubated with the antibody mixture (Table S2) for 30 minutes at room temperature and subsequently washed once with the washing solution. Samples were measured on a BD FACS Canto II flow cytometer. The quality control of the flow cytometers was performed daily and the compensation settings were periodically updated following a standardised protocol.

Table S2. The leukaemic stem cell tube

	Marker	Fluor chrome	Clone
1	CD45	HV500c	2D
2	CD34	BV421	581
3	CD38	APC	HB7
4	CD33	PE-Cy7	P67.6
5	CD44	APC-H7	G44-26
6	CD45RA	FITC	L48
7	CD123	PerCP-Cy5.5	7G3
	Combi		
8	CD7	PE	M-T701
9	CD11b	PE	D12
10	CD22	PE	S-HCL-1
11	CD56	PE	My31
12	CD366 (TIM3)	PE	344823
13	CD371 (Clec12a)	PE	50C1

The 8-colour LSC tube includes common markers (CD45, CD34, CD38) next to lineage- and leukaemia-associated markers. (1) Antigens without expression on normal stem cells are combined within the PE-channel (further referred to as "Combi"), since their cumulative expression on normal stem cells remains negative. CD45RA is absent on normal stem cells but studied separately as this marker was added later on, i.e. after having validated the Combi channel. CD33, CD44 and CD123 are expressed by normal stem cells and should therefore be studied separately to define over- and underexpression. All antibodies were purchased from BD Biosciences, except for the CD366 antibody that was obtained from R&D Systems.

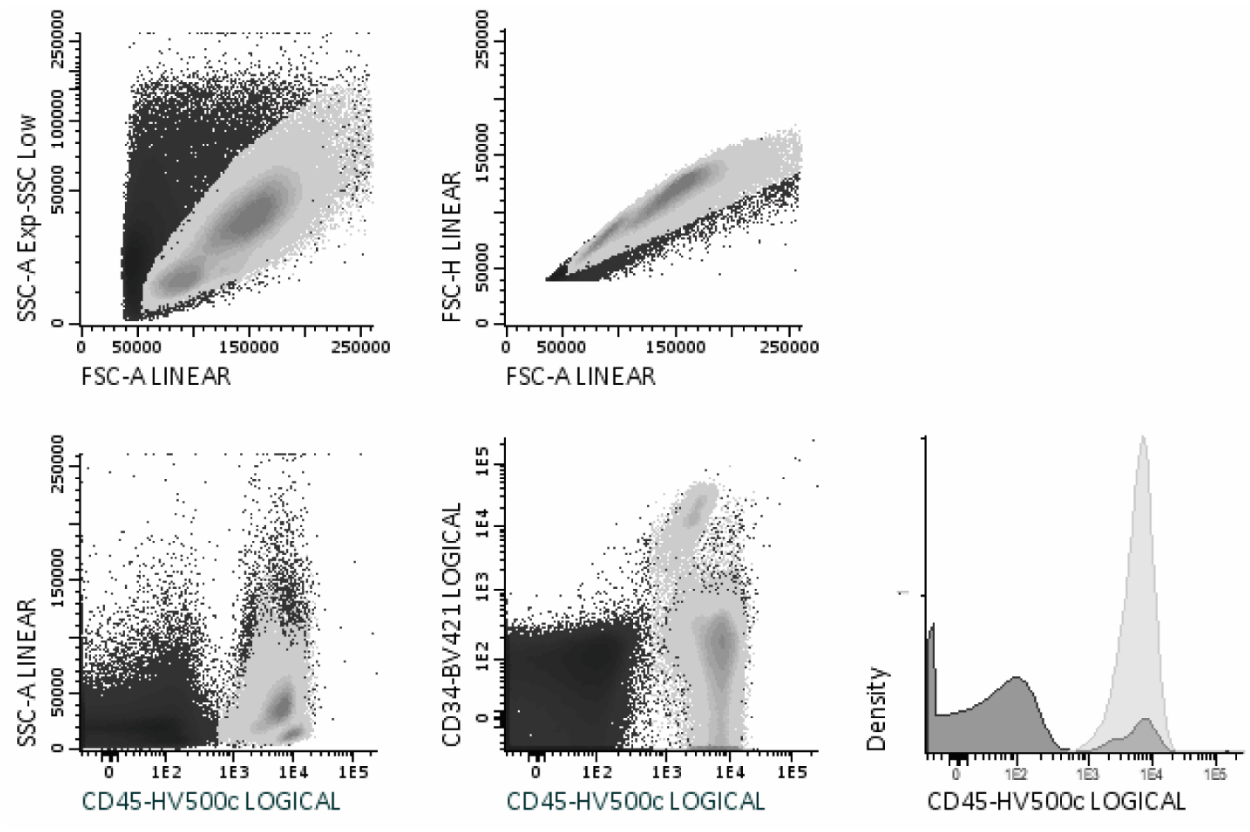

Figure S2. Pre-gating strategy

The FACS plots illustrate the gating strategy of the mononuclear cells (MNC, in light grey). The remaining mature erythrocytes, debris and doublets were excluded in FSC-A/SSC-A and FSC-A/FSC-H plots (upper panel). Remaining $CD45^-$ cells were removed in CD45/SSC-A and CD45/CD34 plots in combination with a CD45 histogram (lower panel).

<u>Data pre-processing</u>

Flow cytometry data were manually pre-gated on MNCs using Infinicyt software 1.8 (Cytognos) (Figure S2). Pre-gated FCS files were subjected to pre-processing steps to remove technical errors potentially hindering biological interpretation. Quality control was performed using the R package flowAI that accounts for abrupt fluctuations in the flow rate, signal acquisition instability, and outliers and margin events. (2) Protein expression data were compensated using the spill-over matrix from the FCS file and transformed using hyperbolic arcsin with a cofactor of 150. A manufacturing change in antibody concentration resulted in a time-related difference in the CD34 expression. To minimise this batch effect and to account for any changes in antibody and scatter intensities over time, range scaling (1 - 99%) between files was performed. The median cell count of all files was $0.83 \cdot 10^6$ pre-gated and pre-processed MNCs per file (Figure S3). Files were randomly subsampled to a maximum of $0.5 \cdot 10^6$ MNCs and aggregated into a dataset of $81 \cdot 10^6$ MNCs from 183 subjects.

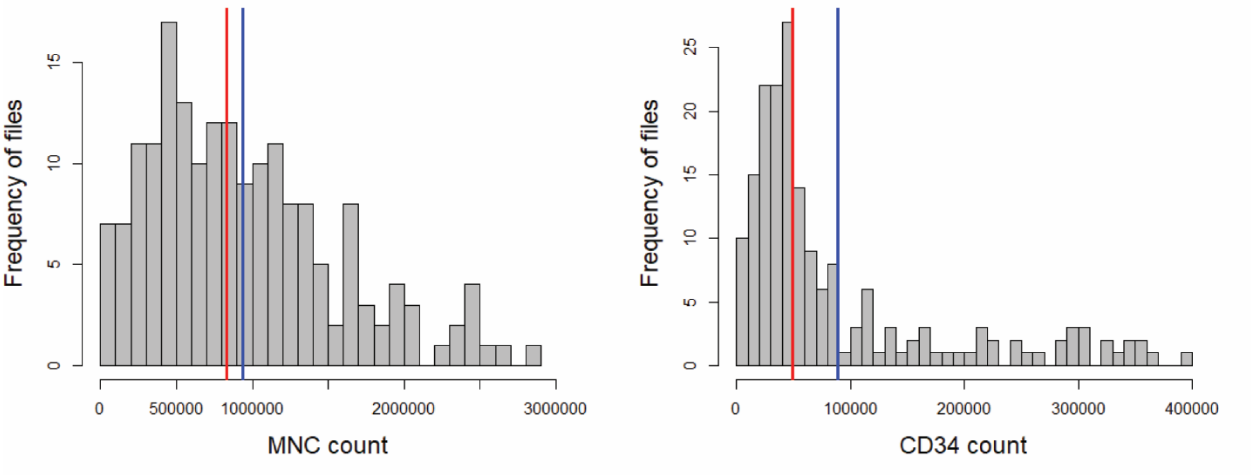

Figure S3. MNC and CD34$^+$ cell counts

The histograms illustrate the counts of the MNC and CD34$^+$ compartments. The MNC counts are derived from manual pre-gating on the pre-processed files. Differently, the CD34$^+$ counts are derived from the computational selection of the CD34 clusters within the FlowSOM tree applied on the MNC compartment. The red and blue lines indicate median and mean values, respectively.

Clustering analysis

The unsupervised algorithm FlowSOM was used to cluster the MNC and CD34 datasets. (3) First, FlowSOM was applied on the aggregated dataset of $81{\cdot}10^6$ manually pre-gated MNCs using CD45, CD34 and the scatter properties as input for cell clustering. The MNC dataset was classified into 25 clusters and 10 metaclusters (or populations). Two CD34$^+$ populations were identified. All cells within the CD34$^+$ metaclusters were selected, ranging from $3.6{\cdot}10^2$ to $2.8{\cdot}10^6$ CD34$^+$ cells (median: $8.3{\cdot}10^5$ CD34$^+$ cells) per sample (Figure S3). The aggregated dataset of $16{\cdot}10^6$ CD34$^+$ cells was subjected to FlowSOM again, discriminating 36 clusters and 25 metaclusters (or HSPC populations) based on all markers apart from the Combi channel. No clustering was observed on potential batch effects including the number of CD34$^+$ cells, used flow cytometer and combined usage of fresh and cryopreserved samples. The moderate clustering over time matched the pattern from AML diagnosis, suggesting that these results were caused by biological signals rather than technical variations over time (Figure S4). The FlowSOM version 1.18.0 was used in R. A general demonstration of the R code of the FlowSOM analysis pipeline is available on GitHub.

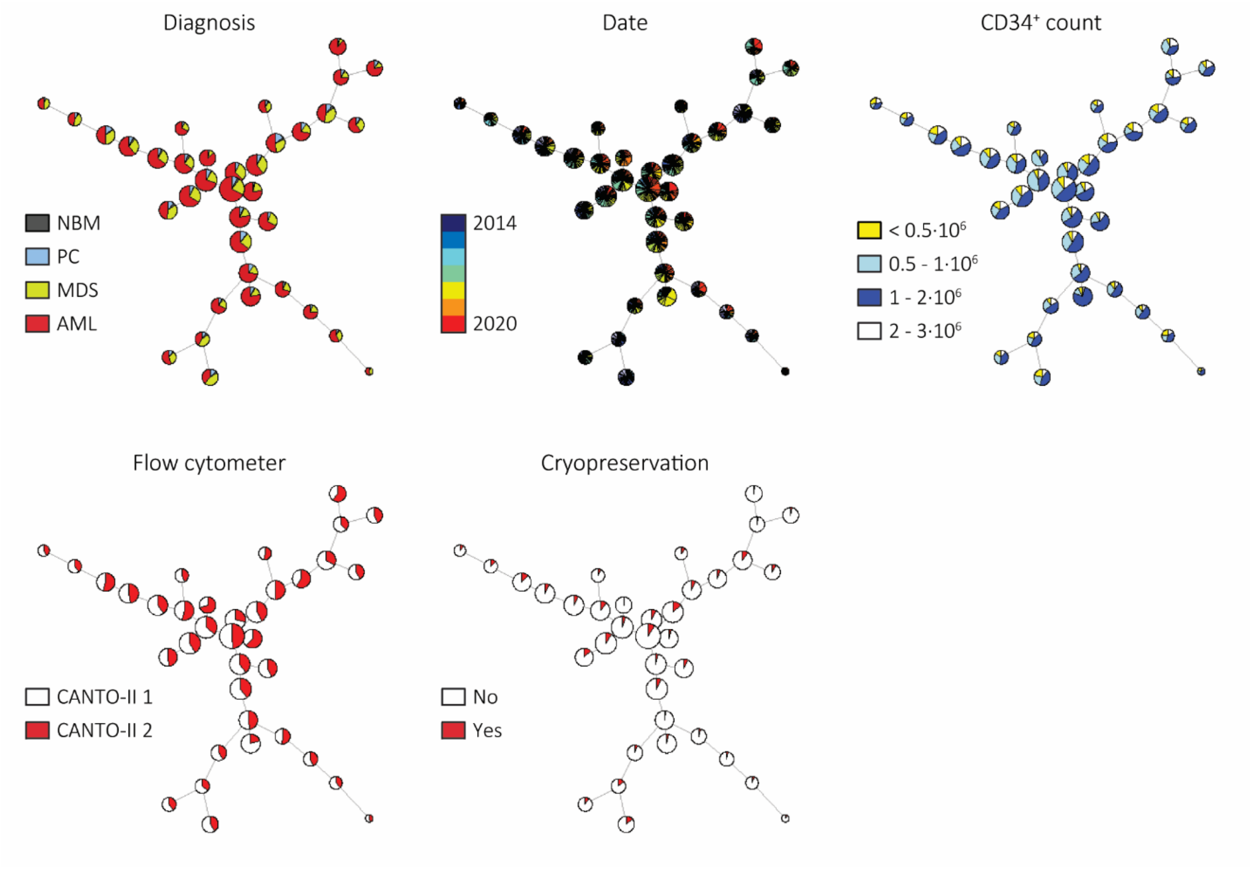

Figure S4. Cell clustering does not reveal potential batch effects

The five $CD34^+$ FlowSOM trees are labelled by diagnosis and potential batch effects. The labels of the $CD34^+$ count, the used flow cytometer and the use of cryopreserved-thawed samples are equally distributed among clusters. Labels indicating the time of experiment match diagnostic patters, suggesting an unequal inclusion of diagnosis over time rather than technical variation.

Cell cluster interpretation

Populations as identified by FlowSOM were labelled quantitatively using Marker Enrichment Modelling (MEM). (4) This metric calculated enrichment scores for each of the protein markers and scatter properties on the 25 cell populations using single-cell data that was clustered by FlowSOM as input. These quantitative labels for each of the populations are relative to all other populations. Besides MEM, populations were labelled manually based on biaxial scatter plots in accordance with the traditional classification for HSPCs, including HSC, CLPs, CMPs, GMPs and MEPs.

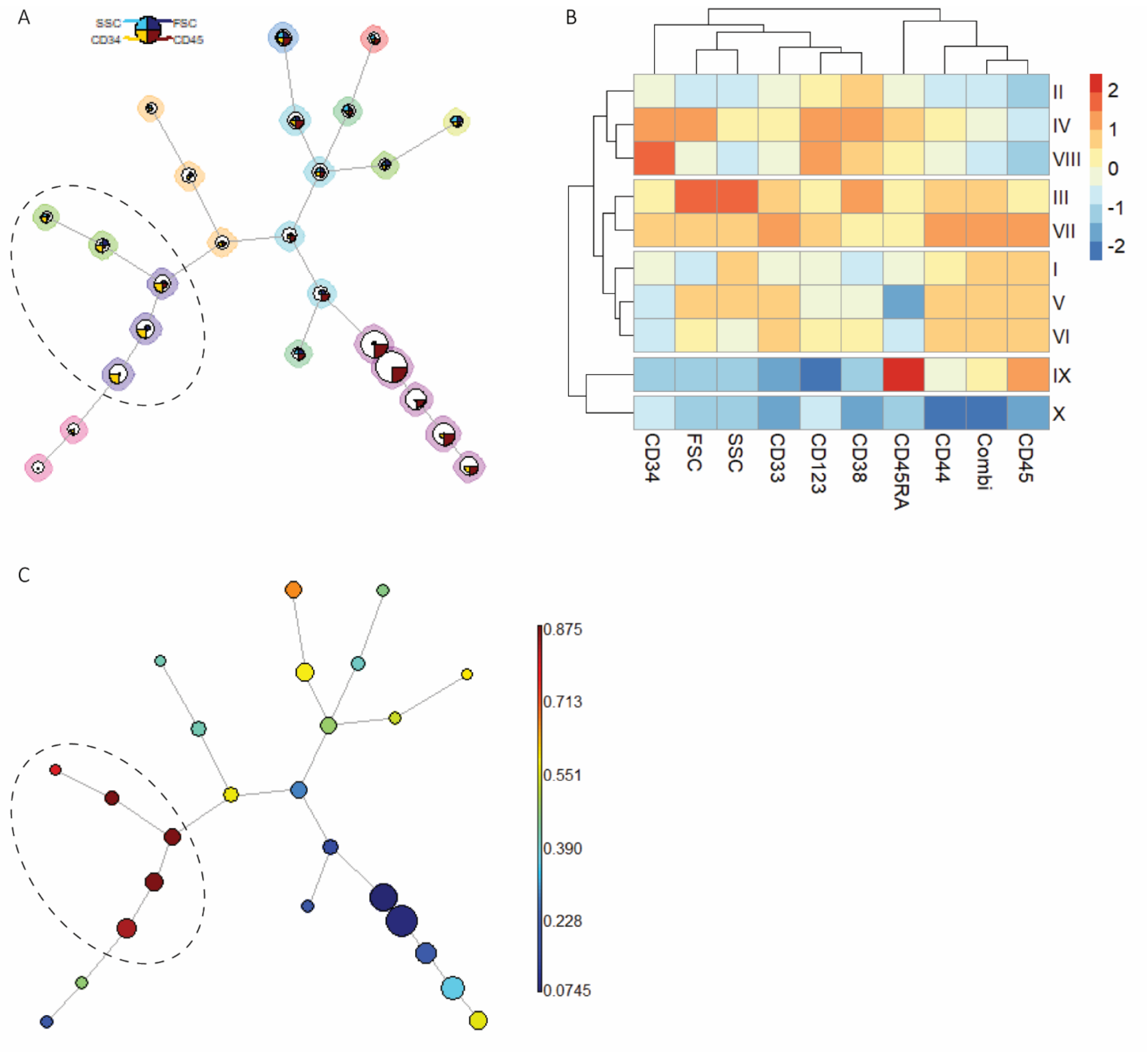

Figure S5. FlowSOM analysis of the MNC compartment

(A) FlowSOM tree of CD34$^+$ cells. The height of the plot pie visualises the expression of the surface markers and the scatter properties. The size of the nodes is proportional to the fraction of cells mapped to the node. Two CD34$^+$ populations [IV, XIII] with high CD34 expression (encircled by the dotted line) were selected and subjected to the second FlowSOM clustering procedure. (B) Heatmap summary of scatter properties and marker expressions for each of the MNC populations. (C) FlowSOM trees coloured by the median expression of CD34.

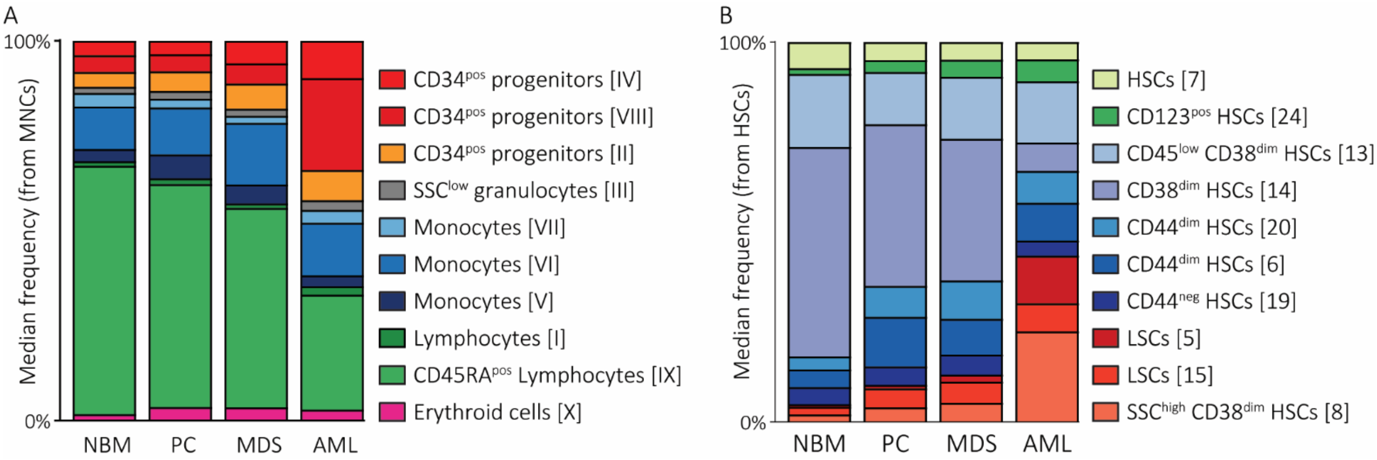

Figure S6. Distribution of MNC and HSC populations over diagnostic groups

(A) Stacked histograms illustrating the median percentages with the 95% confidence interval of the 10 MNC populations (see Figure S5B) relative to the total MNC compartment per diagnosis. (B) Stacked histograms illustrating the median percentages of 10 CD34^{+} populations that were classified as HSC subsets relative to the total HSC compartment per diagnosis. Compared to NBM, PCs show increased CD44^{dim} HSCs [6] , AML samples show increased LSCs [5] and MDS samples show both increased CD44^{dim} HSCs [6] and LSCs [5]. Differences in the relative number of the populations were tested for statistical significance using the Mann-Whitney U test (*P* values incorporated in the result section).

Table S3. Statistical summary of the diagnostic value of CD34$^+$ populations

	Manual Label	NBM vs PC	NBM vs AML	NBM vs MDS	PC vs MDS	AML vs MDS
1	GMPs	ns	ns	0.076	0.026	ns
2	CD44^{dim} CMPs	0.039	0.084	0.060	ns	ns
3	GMPs	ns	ns	ns	ns	<0.001
4	GMPs	0.023	0.024	ns	ns	<0.001
5	LSCs	ns	0.019	ns	ns	0.001
6	CD44^{dim} HSCs	0.050	ns	ns	0.023	<0.001
7	HSCs	ns	0.006	0.047	0.056	0.032
8	SSC^{high} CD38^{dim} HSCs	ns	0.008	ns	ns	<0.001
9	SSC^{low} CMPs	ns	ns	ns	0.008	ns
10	CD34^{dim} prog	ns	0.010	0.054	0.019	<0.001
11	CMPs	ns	0.015	ns	ns	<0.001
12	MDP	ns	<0.001	0.069	ns	<0.001
13	CD45^{low} CD38^{dim} HSCs	0.009	<0.001	0.076	ns	<0.001
14	CD38^{dim} HSCs	0.047	<0.001	0.006	0.014	<0.001
15	LSCs	0.064	ns	ns	ns	0.035
16	CD33$^-$ myeloid prog	ns	0.019	ns	ns	0.003
17	CLPs	ns	<0.001	0.001	<0.001	<0.001
18	CD33^{low} CMPs	ns	ns	ns	ns	ns
19	CD44$^-$ HSCs	ns	<0.001	ns	ns	<0.001
20	CD44^{dim} HSCs	0.045	ns	ns	ns	<0.001
21	CD34^{dim} prog	ns	<0.001	ns	ns	<0.001
22	MEPs	ns	<0.001	ns	0.028	<0.001
23	CD33$^-$ prog	ns	ns	0.096	0.007	ns
24	CD45^{low} HSCs	ns	ns	ns	ns	ns
25	Lymphoid prog	ns	<0.001	<0.001	<0.001	0.033

The table summarises the statistically significant differences in CD34$^+$ population frequencies between diagnostic groups as determined by the Mann-Whitney U test. *P*-values are presented for significant values (P <0.05) and for trends towards significant values (P = 0.050 - 0.100), whereas *P*-values above 0.100 are presented as ns (not significant). Abbreviations: CLPs, common lymphoid progenitors; CMPs, common myeloid progenitors; GMPs, granulocyte-monocyte progenitors; HSCs, hematopoietic stem cell; LSCs, leukaemic stem cells; MEPs, megakaryocyte-erythroid progenitors; MDP, macrophage/dendritic progenitors; prog, progenitors.

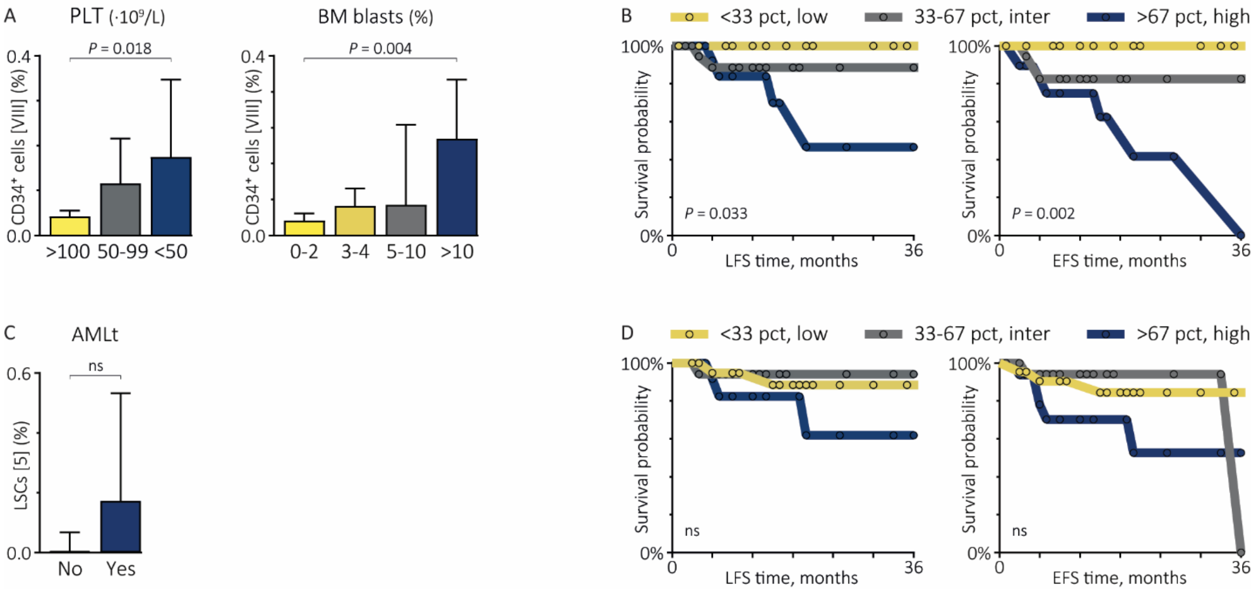

Figure S7. Clinical and prognostic value of CD34⁺ progenitor and LSC frequencies

(A) Relationship between the number of CD34⁺ population [VIII] and platelet levels and BM blast percentages as enumerated by morphology. The CD34⁺ population [VIII] contains CD34⁺ hematopoietic stem and progenitor cells, whereas the blast compartment may also include CD34⁻ cells. Median values with the 95% confidence interval are shown. Differences were tested for statistical significance by the Kruskal-Wallis test. (B) The Kaplan-Meier curves illustrate the prognostic value of the frequency of CD34⁺ population [VIII] stratified into three groups based on the 33rd and 67th percentiles in MDS patients for leukaemic transformation and disease progression. Survival distributions were compared using the log-rank test. (C) MDS patients with transformation towards AML during follow-up appear to have a higher frequency of LSCs [5] at diagnosis than MDS patients with a stable disease, although this difference reached no statistical significance based on the Mann-Witney U test. (D) The Kaplan-Meier curves illustrate the prognostic value of the frequency of LSCs [5] stratified into three groups based on the 33rd and 67th percentiles in MDS patients for leukaemic transformation and disease progression. The log-rank test indicated that the difference between the survival distributions was not statistically significant. Abbreviations: AMLt; transformation towards AML; EFS, event-free survival; LFS, leukaemia-free survival; PLT, platelets

Table S4. Overview of MDS patients with sequential samples

UPN	Label	WHO	Therapy *D sample*	Response *FU sample*	Response *final FU*
MDS03	SD	SF3B1	BSC	SD (8 mo)	MDS-unrelated death (25 mo)
MDS20	SD	LB	LEN, AZA	SD (15 mo)	Alive (46 mo)
MDS04	SD	SF3B1	LEN	SD (14 mo)	PD (64 mo)
MDS27	PD/AML	SF3B1	none	AML (3 mo)	Therapy-related death (8 mo)
MDS28	PD/AML	LB	none	AML (8 mo)	MDS-related death (16 mo)
MDS68	PD/AML	LB	BSC	PD (35 mo)	MDS-related death (40 mo)
MDS73	PD/AML	LB	BSC	PD (3 mo)	MDS-related death
	post CTx		SCT	mCR (5 mo)	
MDS08[1]	post CTx	SF3B1	CTx	no CR (5 mo)	MDS-related death (16 mo)
MDS29	post CTx	SF3B1	CTx	no CR (8 mo)	MDS-related death (10 mo)

[1]: progression to EB-2 after two months. Abbreviations: AZA, azacitidine; BSC, best supportive care; CTx, chemotherapy; D, diagnosis; FU, follow-up; LB, MDS with low blasts; LEN, lenalidomide; mCR, morphological complete remission; no CR, no complete remission; PD, progressive disease; SCT, stem cell transplantation; SD, stable disease; SF3B1, MDS with low blasts and SF3B1 mutation

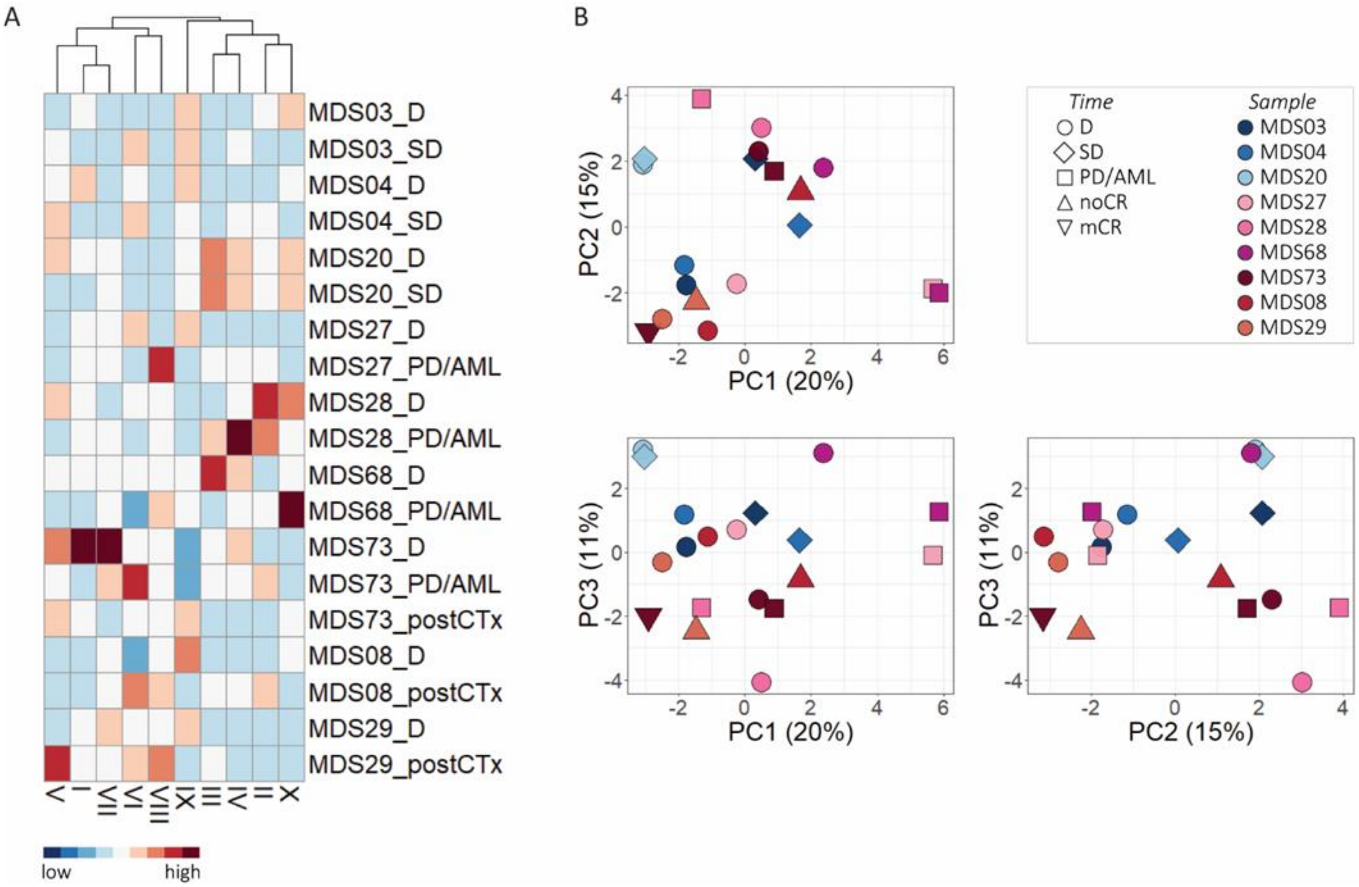

Figure S8. MNC and CD34$^+$ cell compartment changes during follow-up (supplemental to Figure 4)

Samples at time of diagnosis (n = 9) were compared with sequential samples during follow-up (n = 10). From one patient (MDS73), sequential samples were collected during disease progression and after chemotherapy. (A) Heatmap summary of the median percentages of MNC populations at the time of diagnosis and follow-up in MDS patients with a stable disease, disease progression or leukaemic transformation and a residual disease or morphological complete remission after chemotherapy. The MNC populations included progenitors [IV,VIII], CD34$^-$ progenitors [II], SSC^{low} granulocytes [III], monocytes [V-VII], lymphocytes [I,IX] and erythroid cells remaining after cell lysis [X]. (B) A principal component analysis applied on the CD34$^+$ population frequencies for 19 samples derived from 9 MDS patients with a stable disease (n=3), progressive disease or leukemic transformation (n=3) and residual disease or morphological complete remission after chemotherapy (n=3). Only the diagnosis and follow-up samples from MDS20 cluster, whereas the other samples are scattered throughout the plot indicating their changed CD34$^+$ cell composition during the disease course. Note that the sample from patient MDS73, which was considered as a morphological complete remission following chemotherapy despite disease progression afterwards again, does not overlap with the samples from stable disease patients. Abbreviations: FU, follow-up; mCR, morphological complete remission; noCR, no complete remission; PC, principal components; PD/AML, progressive disease or leukemic transformation; postCTx, after cytotoxic therapy (chemotherapy or allogeneic stem cell transplantation); SD, stable disease

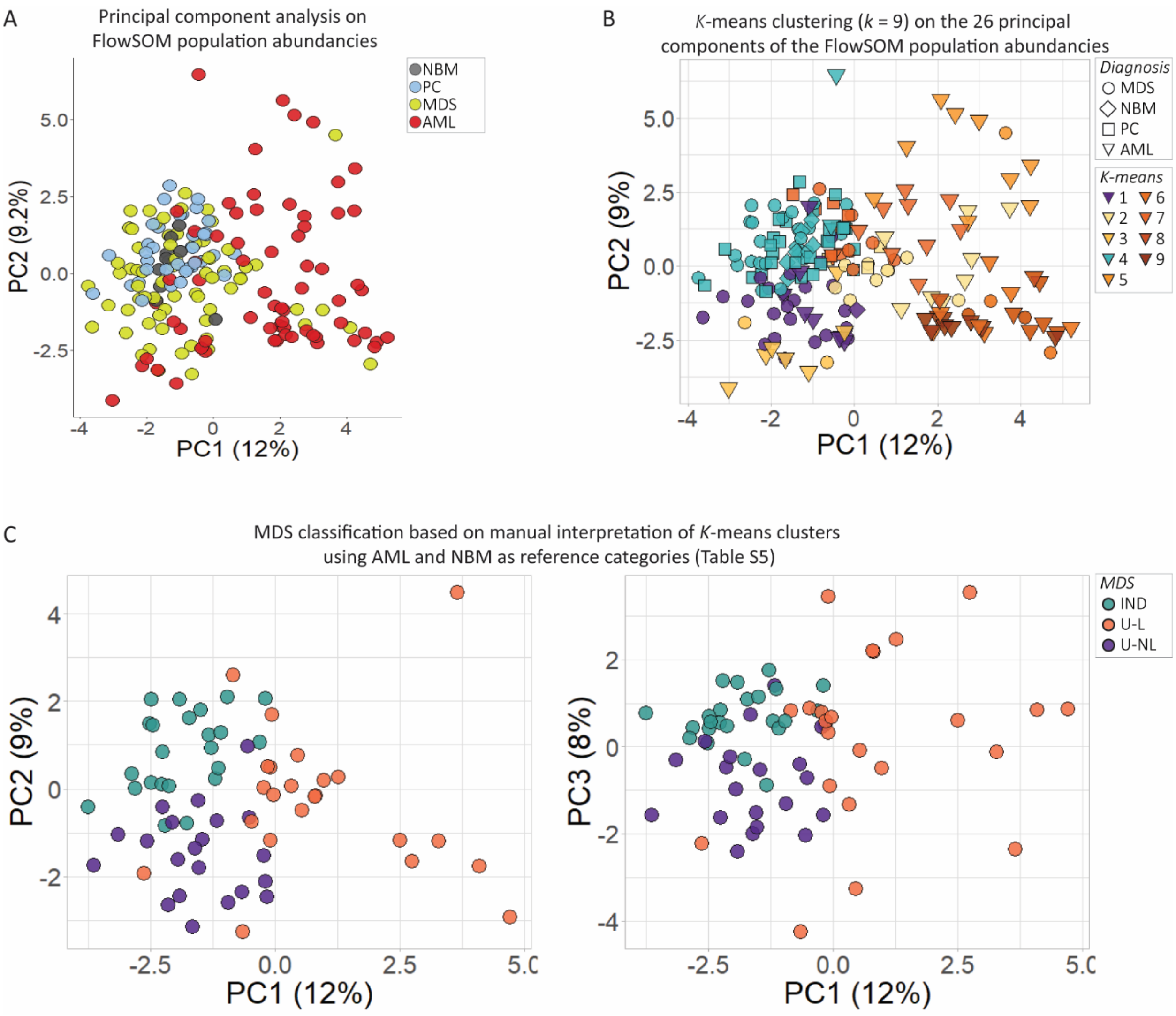

Figure S9. *K*-means clustering on the principal components of the population abundancies.

(A) A principal component analysis applied on the $CD34^+$ population frequencies for each sample. While PCs cluster with NBMs, MDS and AML samples are scattered throughout the plot indicating their variance within the $CD34^+$ cell composition. In contrast to AML samples placed aside from NBMs and PCs, MDS samples overlap with controls and AML samples. (B) *K*-means clustering (k = 9) was applied on the 26 principal components obtained from the principal component analysis of the $CD34^+$ population frequencies. (C) MDS patients were distributed over 8 out of the 9 clusters that were manually classified into 3 subtypes based on the abundance of NBM and AML besides MDS samples within the individual clusters (see Table S5). Note that 3 out of 26 principal components are shown, explaining only 43% of the variance.

Table S5. MDS classification based on *K*-means clustering.

k	Label	MDS, N %	NBM, N %	AML, N %
1	MDS-UN-O	21 (31.1)	2 (20%)	8 (11.9)
2	MDS-UN-L	9 (13.4)	0	8 (11.9)
3	MDS-UN-L	2 (3.0)	0	7 (10.4)
4	MDS-IND	23 (34.3)	8 (80%)	2 (3.0)
5	MDS-UN-L	1 (1.5)	0	9 (13.4)
6	MDS-UN-L	2 (3.0)	0	7 (10.4)
7	MDS-UN-L	7 (10.4)	0	10 (14.9)
8	MDS-UN-L	2 (3.0)	0	7 (10.4)
9	-	0	0	9 (13.4)

K-means clustering with k = 9 was applied on the 36 principal components of the frequencies of the FlowSOM populations (see Figure S9). Using NBM and AML samples as reference categories, the clusters with MDS patients (excluding cluster 9) were classified as MDS-IND (indolent), MDS-UN-L (unfavourable, leukaemic), and MDS-UN-O (unfavourable, other). The percentages are proportional to the diagnostic group.

Table S6. Statistical summary of the differences in $CD34^+$ populations between MDS subtypes

	Manual Label	IND vs UN-L & UN-O	UN-L IND & UN-O	UN-O IND & UN-L
1	GMPs	ns	0.004	0.081
2	$CD44^{dim}$ CMPs	ns	0.004	<0.001
3	GMPs	0.081	<0.001	0.017
4	GMPs	0.014	0.022	ns
5	LSCs	0.005	0.043	ns
6	$CD44^{dim}$ HSCs	ns	ns	ns
7	HSCs	ns	ns	ns
8	SSC^{high} $CD38^{dim}$ HSCs	ns	ns	ns
9	SSC^{low} CMPs	<0.001	0.057	<0.001
10	$CD34^{dim}$ prog	ns	ns	ns
11	CMPs	ns	<0.001	<0.001
12	MDP	0.043	ns	0.001
13	$CD45^{low}$ $CD38^{dim}$ HSCs	ns	<0.001	<0.001
14	$CD38^{dim}$ HSCs	<0.001	0.002	ns
15	LSCs	ns	ns	ns
16	$CD33^{-}$ myeloid prog	0.004	0.052	ns
17	CLPs	<0.001	0.027	0.011
18	$CD33^{low}$ CMPs	ns	ns	ns
19	$CD44^{-}$ HSCs	ns	ns	ns
20	$CD44^{dim}$ HSCs	ns	ns	ns
21	$CD34^{dim}$ prog	<0.001	0.002	0.084
22	MEPs	<0.001	<0.001	ns
23	$CD33^{-}$ prog	0.081	ns	ns
24	$CD45^{low}$ HSCs	0.014	0.034	ns
25	Lymphoid prog	<0.001	0.002	0.037

The table summarises the statistically significant differences in $CD34^+$ population frequencies as determined by the Mann-Whitney U test between MDS subgroups based on *K*-means clustering (Table S5). *P*-values are presented for significant values (P <0.05) and for trends towards significant values (P = 0.050 - 0.100), whereas *P*-values above 0.100

are presented as ns (not significant). Abbreviations: CLPs, common lymphoid progenitors; CMPs, common myeloid progenitors; GMPs, granulocyte-monocyte progenitors; HSCs, hematopoietic stem cell; IND, indolent subtype; LSCs, leukaemic stem cells; MDP, macrophage/ dendritic progenitor; MEPs, megakaryocyte-erythroid progenitors; prog, progenitors; UN-L, unfavourable, leukaemic subtype; UN-O, unfavourable, other subtype

References

1. Zeijlemaker W, Kelder A, Oussoren-Brockhoff YJ, Scholten WJ, Snel AN, Veldhuizen D, *et al*. A simple one-tube assay for immunophenotypical quantification of leukemic stem cells in acute myeloid leukemia. *Leukemia* 2016; 30: 439-46.

2. Monaco G, Chen H, Poidinger M, Chen J, de Magalhães JP, Larbi A. flowAI: automatic and interactive anomaly discerning tools for flow cytometry data. *Bioinformatics* 2016; 32: 2473-80.

3. Van Gassen S, Callebaut B, Van Helden MJ, Lambrecht BN, Demeester P, Dhaene T, *et al*. FlowSOM: Using self-organizing maps for visualization and interpretation of cytometry data. *Cytometry A* 2015; 87: 636-45.

4. Diggins KE, Greenplate AR, Leelatian N, Wogsland CE, Irish JM. Characterizing cell subsets using marker enrichment modeling. *Nat Methods* 2017; 14: 275-8.
